# Regulation of lipid droplets by metabolically controlled Ldo isoforms

**DOI:** 10.1083/jcb.201704115

**Published:** 2018-01-02

**Authors:** Vitor Teixeira, Lisa Johnsen, Fernando Martínez-Montañés, Alexandra Grippa, Laura Buxó, Fatima-Zahra Idrissi, Christer S. Ejsing, Pedro Carvalho

**Affiliations:** 1 Sir William Dunn School of Pathology, University of Oxford, Oxford, England, UK; 2 Cell and Developmental Biology Programme, Centre for Genomic Regulation, Barcelona, Spain; 3 Universitat Pompeu Fabra, Barcelona, Spain; 4 Department of Biochemistry and Molecular Biology, Villum Center for Bioanalytical Sciences, University of Southern Denmark, Odense, Denmark

## Abstract

Storage and consumption of neutral lipids in lipid droplets (LDs) are essential for energy homeostasis and tightly coupled to cellular metabolism. However, how metabolic cues are integrated in the life cycle of LDs is unclear. In this study, we characterize the function of Ldo16 and Ldo45, two splicing isoforms of the same protein in budding yeast. We show that Ldo proteins interact with the seipin complex, which regulates contacts between LDs and the endoplasmic reticulum (ER). Moreover, we show that the levels of Ldo16 and Ldo45 depend on the growth stage of cells and that deregulation of their relative abundance alters LD morphology, protein localization, and triglyceride content. Finally, we show that absence of Ldo proteins results in defects in LD morphology and consumption by lipophagy. Our findings support a model in which Ldo proteins modulate the activity of the seipin complex, thereby affecting LD properties. Moreover, we identify ER–LD contacts as regulatory targets coupling energy storage to cellular metabolism.

## Abstract (precis)

Lipid droplets (LDs) are essential organelles for cellular energy homeostasis, but how metabolic cues are integrated in their life cycle is unclear. Teixeira et al. find that two protein isoforms, Ldo16 and Ldo45, differentially regulate LD dynamics under nutrient-rich and -deprived conditions, linking energy metabolism and storage.

## Introduction

Cells live in fluctuating environments, and the capacity to store energy during nutrient surplus to mobilize upon deprivation is essential for their survival. In eukaryotic cells, energy is accumulated in the form of highly reduced neutral lipids, mainly triglycerides (TAGs) and steryl esters (SEs), in dedicated organelles called lipid droplets (LDs; Walther and Farese, 2012; Wang, 2015). LD deregulation is associated with common diseases such as lipodystrophy, metabolic syndrome, and atherosclerosis, highlighting their central role in energy homeostasis (Krahmer et al., 2013). However, how metabolic cues regulate LD growth and consumption is largely unknown.

LDs consist of neutral lipids in their core surrounded by a phospholipid monolayer and specific proteins essential in regulating their growth and consumption (Yang et al., 2012). During their life cycle, most LDs remain associated with the ER through physical contacts stabilized by the Fld1–Ldb16 seipin complex (Szymanski et al., 2007; Fei et al., 2008; Jacquier et al., 2011; Cartwright et al., 2014; Wang et al., 2014a, 2016; Grippa et al., 2015; Salo et al., 2016). Although seipin mutants still form LDs, they exhibit defective morphology and composition caused by aberrant ER–LD contacts. The precise mechanisms by which seipin stabilizes ER–LD contacts are unclear, but they may establish a diffusion barrier either through oligomerization (Binns et al., 2010) or local remodeling of the lipid environment (Fei et al., 2011; Han et al., 2015; Wolinski et al., 2015; Pagac et al., 2016). Moreover, it is not known how seipin is regulated in response to metabolic cues. ER–LD contacts are critical during LD biogenesis, appearing as attractive regulatory targets to couple cellular metabolism to energy storage. In this study, we identify the proteins LD Organization 16KDa (Ldo16) and LD Organization 45KDa (Ldo45) as ancillary subunits of the seipin complex. Interestingly, these are encoded by the same gene and result from a splicing event. We show that Ldo isoforms are differentially expressed depending on the cellular metabolic status and have distinct effects on LDs. Although Ldo45 promotes LD proliferation and TAG accumulation, Ldo16 is necessary for efficient LD consumption by lipophagy upon starvation. These findings indicate that Ldo proteins partner with seipin to couple regulation of ER–LD contacts with cellular metabolic states.

## Results and discussion

### Two Ldo isoforms interact with the seipin complex

To identify putative regulators of the yeast Fld1–Ldb16 seipin complex, we immunoprecipitated endogenous TAP-tagged Fld1 and Ldb16, and copurifying proteins were analyzed by mass spectrometry. Both purifications identified two proteins encoded by adjacent ORFs: *YMR147W* and *YMR148W*.

**Figure 1.**
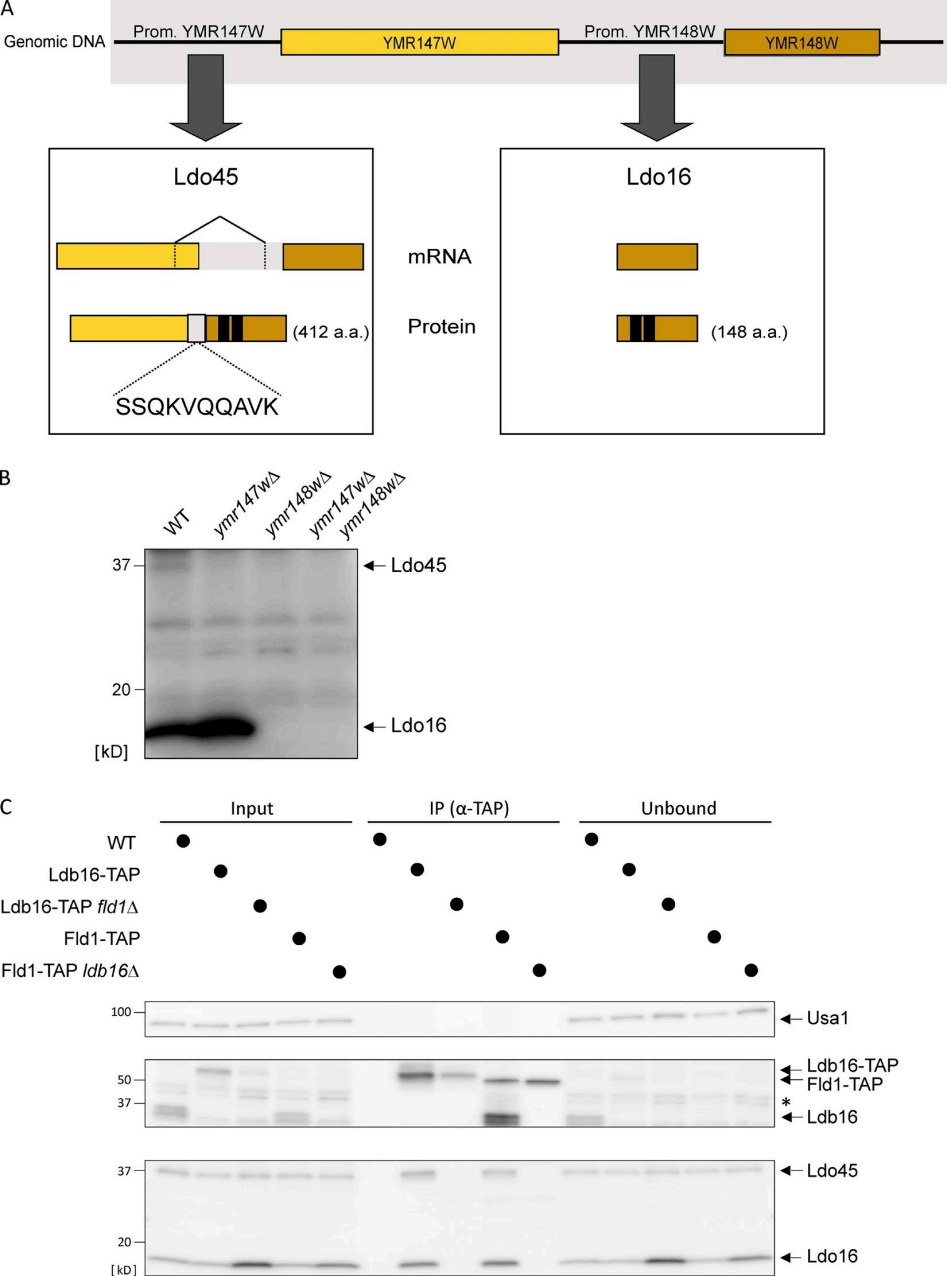
**Two Ldo isoforms interact with the seipin complex. (A)** Scheme of *YMR147W* and *YMR148W* loci. The boxes indicate gene products from transcription through *YMR147W* (left) or *YMR148W* (right) promoters and that give rise to Ldo45 and Ldo16 proteins, respectively. The amino acid number of each protein is in parenthesis. In the left box, the splicing event giving rise to Ldo45 is depicted. Among the Ldo45 peptides detected by mass is one encoded by the intragenic regions (in light gray), confirming this splicing event. In black is the predicted hydrophobic hairpin involved in membrane association of both Ldo proteins. **(B)** Protein extracts from cells with the indicated genotype were analyzed by Western blotting with polyclonal antibody-recognizing epitopes on the C termini common to Ldo45 and Ldo16. **(C)** Ldo45 and Ldo16 coprecipitate with TAP-tagged Ldb16 and Fld1. Detergent-solubilized extracts prepared from cells with the indicated genotype were immunoprecipitated (IP), and eluted proteins were analyzed by Western blotting. Please note that the TAP-tagged proteins are recognized by the anti-Ldb16 rabbit IgG. The asterisk indicates a nonspecific band.

Earlier transcriptomic studies revealed a curious relationship between these ORFs, with the splicing of an intron at the 3′ region of *YMR147W* generating a hybrid transcript with *YMR148W* (Fig. 1 A; Miura et al., 2006; Schreiber et al., 2015). Transcripts encoding exclusively *YMR148W* were also abundantly detected, suggesting that the two transcripts originate from distinct promoters (Miura et al., 2006). We confirmed the existence of both transcripts using reverse transcription followed by DNA sequencing (unpublished data). We found that splicing excluded the sequence coding for the last 29 amino acids of *YMR147W* and included a 210-bp intragenic region 5′ of *YMR148W* ORF (Fig. 1 A). Using antibodies directed to an HA tag on the Ymr148w C terminus or to Ymr148w itself, we found that both transcripts are translated into proteins of 412 and 148 amino acids (Fig. 1 B). In agreement with Eisenberg-Bord et al., which reports similar findings, we named these proteins Ldo45 and Ldo16, respectively. Ldo16 levels are similar in WT and *ldo45Δ* cells, indicating that Ldo16 expression is driven from its own promoter. Finally, reanalysis of the mass spectrometry data revealed peptides from both Ldo isoforms, including a peptide encoded by the intragenic region demonstrating the splicing event described (Fig. 1 A).

The proteomics results were confirmed by immunoprecipitation of Fld1- and Ldb16-TAP followed by Western blotting. Although Ldo proteins coprecipitated with seipin under various conditions, other abundant ER and LD proteins did not (Figs. 1 C and S1 A). Similarly, Fld1 and Ldb16 coprecipitated with endogenously HA-tagged Ldo proteins (Fig. S1 B). Thus, Ldo proteins interact specifically with seipin complex (Pagac et al., 2016; Eisenberg-Bord et al., 2018). Moreover, Ldo proteins coprecipitated only with an intact seipin core complex as shown by the loss of interaction in the absence of either Fld1 or Ldb16 (Figs. 1 C and S1 B). Only a fraction of Ldo proteins appears to associate with the seipin core components, suggesting that they are ancillary subunits of the complex (Figs. 1 C and S1, A and B). In agreement, Ldo16 (Grillitsch et al., 2011; Grippa et al., 2015; Moldavski et al., 2015) and Ldo45 (Fig. S1 C) have dual localization to the ER and LDs. This is consistent with both protein isoforms sharing a predicted membrane hairpin (Fig. 1 A), a motif allowing association with both ER bilayers and LD monolayers (Pol et al., 2014; Kory et al., 2016).

### Specific effects of Ldo45 in the LD proteome

Seipin has a central role in controlling the LD proteome (Wang et al., 2014b, 2016; Grippa et al., 2015; Salo et al., 2016). To test whether Ldo proteins also impact LD protein targeting, we analyzed LDs isolated from *ldo45Δ ldo16Δ* cells by label-free quantitative proteomics as previously described (Grippa et al., 2015). In contrast with *fld1Δ* and *ldb16Δ*, LDs isolated from *ldo45Δ ldo16Δ* had a proteome largely similar to the control (Fig. S2 A). This was confirmed by fluorescence microscopy of representative LD proteins such as Erg6, Pet10, and Tgl1 (Fig. S2 B and unpublished data).

**Figure 2.**
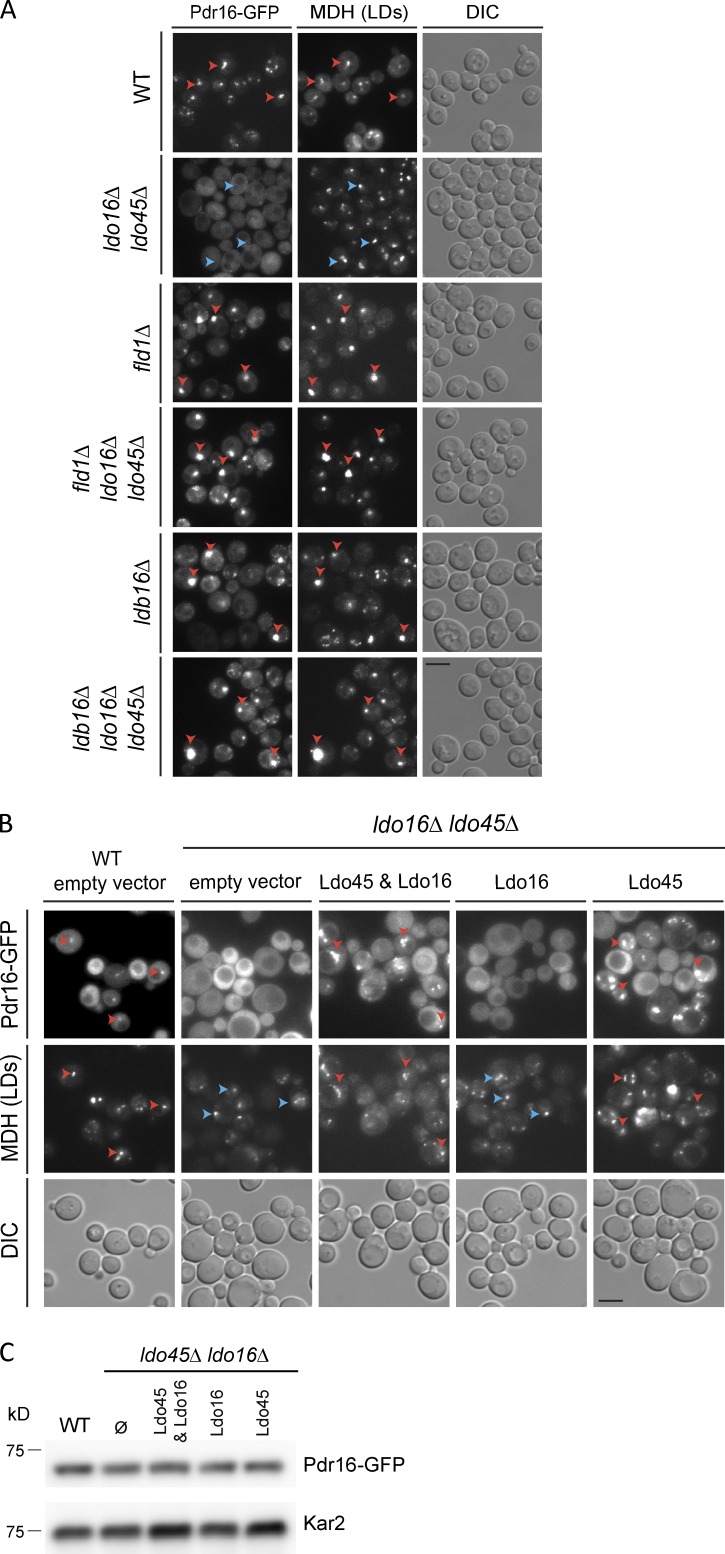
**Specific role of Ldo45 in LD recruitment of Pdr16. (A)** Localization of Pdr16-GFP in cells with the indicated genotype. Cells were grown in YPD to early stationary phase. Red and blue arrowheads indicate existence or absence of colocalization, respectively. **(B)** Localization of Pdr16-GFP in cells with the indicated genotype. Cells were grown in SC media to exponential phase and analyzed as in A. Existence or absence of colocalization is indicated as in A. Bars, 2 µm. DIC, differential interference contrast. **(C)** Pdr16-GFP levels in cells with the indicated genotype. Cells were grown as in B, and protein extracts were analyzed with anti-GFP.

Strikingly, the lipid transfer protein Pdr16/Sfh3 was markedly reduced in LDs from *ldo45Δ ldo16Δ* cells (Fig. S2 A). Consistently, in *ldo45Δ ldo16Δ* mutant, Pdr16-GFP was diffuse throughout the cytoplasm (Fig. 2, A and B; Eisenberg-Bord et al., 2018), whereas in WT cells, it localized to the surface of LDs and the cell periphery, as described previously (Ren et al., 2014). The levels of Pdr16-GFP were similar in *ldo45Δ ldo16Δ* and WT, indicating that the mutant is defective in targeting Pdr16 to LDs (Fig. 2 C).

Ldo proteins fail to localize properly to LDs in seipin mutants (Grippa et al., 2015). Thus, it was puzzling that LDs isolated from *fld1Δ* and *ldb16Δ* contained normal Pdr16 content (Fig. S2 A). In agreement with the mass spectrometry data, Pdr16-GFP was detected not only in LD aggregates of *fld1Δ* and *ldb16Δ* cells but also in *ldo45Δ ldo16Δ fld1Δ* and *ldo45Δ ldo16Δ ldb16Δ*, which lack Ldo proteins (Fig. 2 A). Thus, although necessary for Pdr16 LD recruitment in WT cells, Pdr16 is recruited to LDs independently of Ldo proteins in seipin mutants, suggesting that Ldo proteins do not function as Pdr16 receptors. Indeed, we failed to detect biochemical interactions between Pdr16 and Ldo proteins or any other seipin complex component (unpublished data). We favor a model in which Ldo proteins facilitate Pdr16 recruitment to LDs by regulating their surface properties through the seipin complex.

Next, we tested the contribution of each Ldo isoform to the recruitment of Pdr16 to LDs. Plasmid-borne expression of both Ldo isoforms simultaneously restored Pdr16-GFP LD localization in *ldo45Δ ldo16Δ* cells as expected. Remarkably, Pdr16-GFP LD localization was restored by expression of Ldo45 alone but not by endogenous or overexpressed Ldo16 (Figs. 2 B and S2, C–E; Eisenberg-Bord et al., 2018), suggesting nonredundant functions of Ldo isoforms in LD regulation. The localization differences were not a result of decreased Pdr16 levels (Figs. 2 C and S2 D). Thus, Ldo45 displays an exclusive role in promoting Pdr16 LD recruitment. Pdr16 was previously shown to modulate LD lipolysis (Ren et al., 2014), sensitivity to azole compounds (Holič et al., 2014; Ren et al., 2014), and clearance of certain protein aggregates (Moldavski et al., 2015). Whether and how these different phenotypes are related with the Ldo45-dependent LD recruitment is not clear and should be addressed in future studies.

### Relative abundance of Ldo45 and Ldo16 is metabolically controlled

**Figure 3.**
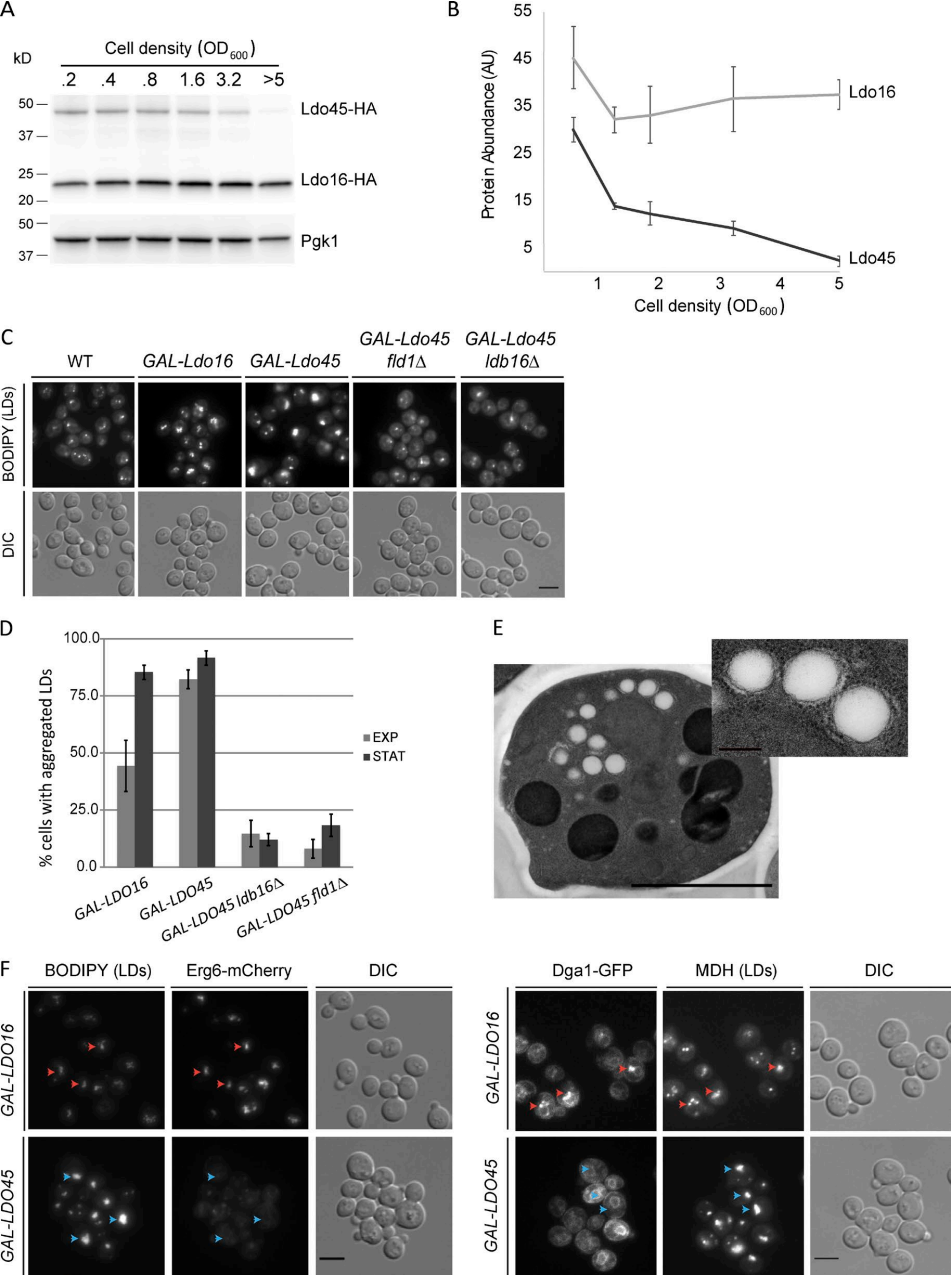
**Metabolic regulation of Ldo45 is required for LD morphology and protein targeting. (A)** Ldo45 levels drop in stationary phase. Protein extracts from WT cells growing in SC medium at the indicated density were analyzed by Western blotting with anti-HA antibodies. **(B)** Quantification of Ldo45 and Ldo16 levels in WT cells analyzed as in A. The graph is the mean of three independent experiments. **(C)** LD morphology in cells overexpressing the indicated Ldo protein from the *GAL1* promoter. **(D)** Quantification of LD aggregates in cells with the indicated genotype grown as in C. Quantifications are from three independent experiments for exponential (EXP) and stationary (STAT) phases, respectively. Error bars indicate SD. **(E)** Thin-section electron micrographs of cells expressing Ldo45 from the strong *GPD* promoter grown in YPD. **(F)** Cell overexpressing the indicated Ldo protein and endogenous Erg6-Cherry (left) or Dga1-GFP (right) were grown as in C for 16 h. Red and blue arrowheads indicate existence or absence of colocalization, respectively. Bars: (main images) 2 µm; (E, inset) 200 nm. DIC, differential interference contrast.

Transcriptional analysis suggested that Ldo16 and Ldo45 expression is controlled by independent promoters (Miura et al., 2006). To test whether the two isoforms were differentially regulated, we analyzed their relative abundance during different growth phases. Remarkably, although Ldo16 protein levels were relatively constant, the amount of Ldo45 dropped gradually but drastically as cells approached stationary phase (Fig. 3, A and B). Replacement of endogenous *YMR147W*/*LDO45* promoter led to comparable levels of Ldo45 in exponential and stationary cells (Fig. S3 A and unpublished data). These results indicate that the drop in Ldo45 levels in stationary phase is not caused by changes in splicing, RNA processing, or protein degradation but likely by reduced activity of the *YMR147W* promoter in stationary phase cells. Thus, Ldo45 levels are tightly coupled to cellular metabolism.

### Ldo45 overexpression affects LD morphology and TAG accumulation

To study the impact of deregulating Ldo45 levels, we replaced *YMR147W*/*LDO45* promoter by the strong glyceraldehyde-3-phosphate dehydrogenase (*GPD*) or galactokinase (*GAL1*) promoters for constitutive or inducible expression, respectively (Janke et al., 2004). In either case, Ldo45 overexpression triggered dramatic proliferation of LDs, which almost invariably clumped into a single aggregate (Fig. 3, C and D; and Fig. S3 B). Although morphologically distinct from aggregates of *fld1Δ* and *ldb16Δ* cells (Wolinski et al., 2011; Grippa et al., 2015), the cross sections of the LDs were frequently wrinkled and irregular, defects also observed in seipin mutants (Fig. 3 E; Grippa et al., 2015). The effects on LD morphology correlated with Ldo45 levels and the presence of Ldo45 binding partners Fld1 and Ldb16 but not with the growth stage (Fig. 3 D). Overexpression of Ldo16 also induced changes in LD distribution; however, they were not as dramatic, having smaller aggregates and only detectable upon prolonged overexpression (Fig. 3, C and D; and Fig. S3 B). Moreover, overexpression of Ldo45 and Ldo16 had distinct effects on the LD protein targeting. Although Erg6, Dga1, and Pet10 localized normally upon Ldo16 overexpression, increased Ldo45 expression prevented their LD targeting (Fig. 3 F and unpublished data). Thus, the two Ldo isoforms have distinct effects on LDs, with Ldo45 specifically stimulating LD aggregation and inducing LD protein mistargeting. Given that both Ldo isoforms associate with membranes through the same hairpin domain (Fig. 1 A) and are overexpressed to comparable levels (Fig. S2 D), it is unlikely that molecular crowding effects, shown to affect the localization of proteins to LDs (Kory et al., 2015), are responsible for the targeting defects specific to Ldo45 overexpression.

**Figure 4.**
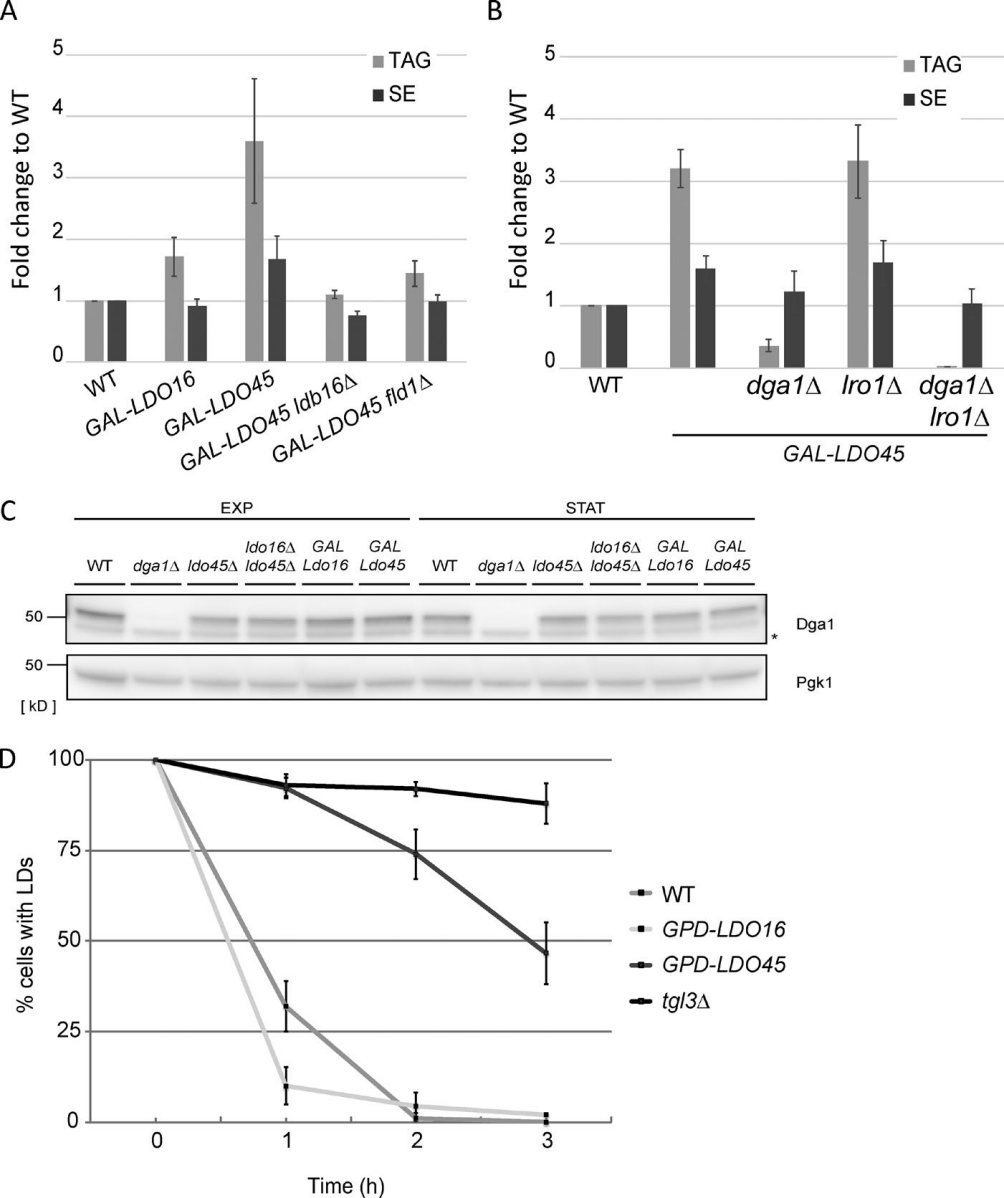
**Deregulation of Ldo45 levels promotes Dga1-dependent TAG accumulation. (A)** Quantification of neutral lipids in cells with the indicated genotype. Cells in YPGal were diluted to OD_600_ 0.1 and grown for 24 h in the presence of 1 µCi/ml^[1–14C]^ acetate. Neutral lipids were extracted and separated by thin layer chromatography. Graphs show the mean of two independent experiments. **(B)** Quantification of neutral lipids in cells with the indicated genotype as in A. **(C)** Protein extracts from cells grown as in Fig. 3 D analyzed by Western blotting with anti-Dga1 antibodies. The asterisk indicates a nonspecific band. EXP, exponential; STAT, stationary. **(D)** LD consumption in cells with the indicated genotype grown in YPD media to late logarithmic phase. Cerulenin (5 µg/ml) was added at time 0 to stimulate LD consumption. The graph displays the mean of three independent experiments. Error bars indicate SD.

LD aggregates induced by Ldo45 overexpression were more prominently labeled by neutral lipid dyes such as BODIPY or monodansyl pentane (MDH; Fig. 3, C and F; and Fig. S3 B). Thus, we tested whether this would reflect changes in neutral lipid content. Indeed, overexpression of Ldo45 resulted in a three- to fourfold increase in TAG levels in comparison with WT cells with no significant effects on SEs (Fig. 4 A). However, TAG accumulation was abrogated in *fld1Δ* and *ldb16Δ* cells expressing similar levels of Ldo45 (Figs. 4 A and S3 A). As in the case of LD morphology, Ldo16 overexpression had only a minor effect on TAG accumulation. Two enzymes, Dga1 and Lro1, are responsible for virtually all TAG synthesis in *Saccharomyces cerevisiae* (Oelkers et al., 2000, 2002). Therefore, we evaluated their contribution to TAG accumulation upon Ldo45 overexpression. Under these conditions, *lro1Δ* accumulated TAG to levels comparable with controls (Fig. 4 B), whereas in *dga1Δ* cells, TAG accumulation was strongly blunted. Thus, Dga1 is largely responsible for the TAG surplus in Ldo45-overexpressing cells. Under these conditions, Dga1 levels remained constant (Fig. 4 C), suggesting that its accumulation in the ER (Fig. 3 F) favors increased TAG synthesis. Indeed, previous studies suggest that Dga1 acyltransferase activity is regulated at multiple levels and depends on its localization as well as accessibility to the substrate diacylglycerol (Oelkers et al., 2002; Dubots et al., 2014; Markgraf et al., 2014).

TAG accumulation in Ldo45-overexpressing cells could also result from impaired lipolysis. To test this hypothesis, we analyzed LD consumption upon stimulation of lipolysis with cerulenin, an inhibitor of fatty acid synthesis. This treatment triggers rapid consumption of LDs in WT cells but not in the *tgl3Δ* mutant lacking the major TAG lipase (Athenstaedt and Daum, 2003). Importantly, overexpression of Ldo45 but not of Ldo16 strongly delayed lipolysis (Fig. 4 D). Thus, both Dga1-dependent synthesis and slowed lipolysis contribute to TAG accumulation upon Ldo45 overexpression.

### Ldo proteins are required for normal LD morphology and lipophagy

**Figure 5.**
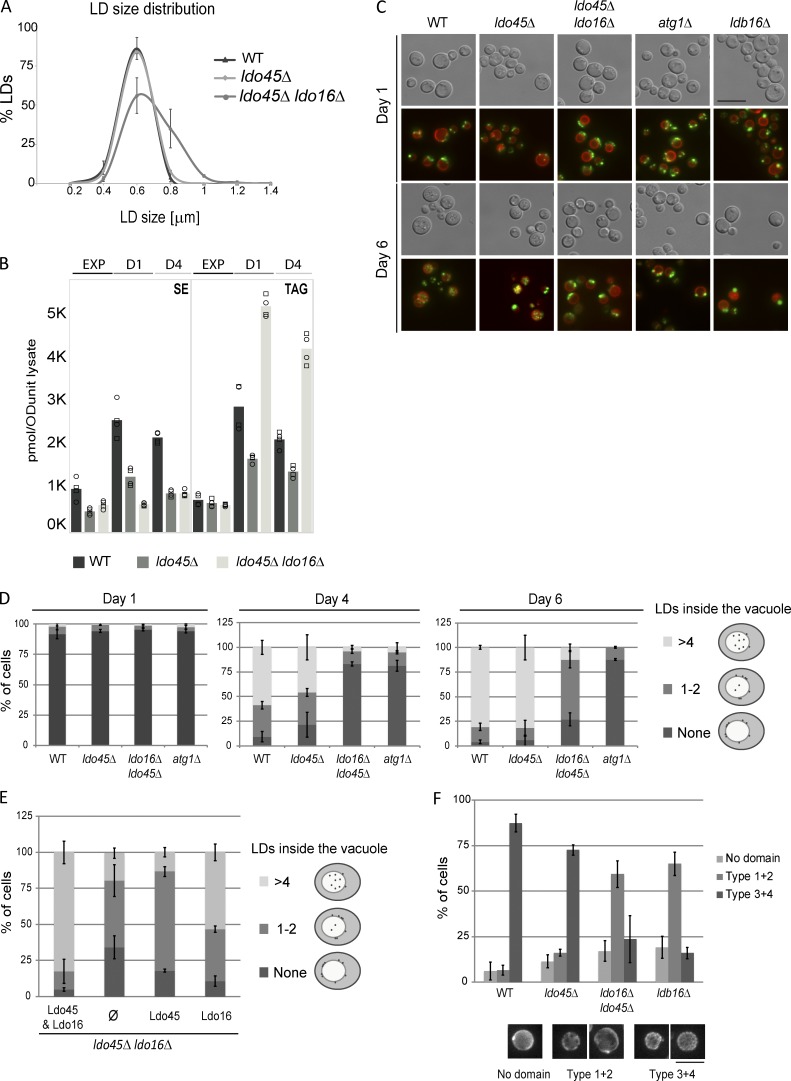
**Ldo proteins are required for normal LD morphology and lipophagy. (A)** Size distribution of LDs of early stationary phase cells with the indicated genotype. **(B)** Levels of SE (left) and TAG (right) in cells with the indicated genotypes grown in SC medium to exponential (EXP), early (day 1; D1), and late stationary (day 4; D4) phases. The graph corresponds with the mean of two biological and two technical repeats. **(C)** Distribution of BODIPY-stained LDs (green) in cells with the indicated genotypes expressing endogenous Vph1-tdTomato (red) as described previously (Wang et al., 2014a). **(D)** Quantification of LDs localized to the vacuole in cells grown as in C at the indicated days. **(E)** Quantification of LDs localized to the vacuole in *ldo16*Δ*ldo45*Δ cells expressing the indicated plasmid-borne Ldo proteins and analyzed as in D. **(F)** Quantification of Lo domains at day 4 in cells with the indicated genotype grown as in C and expressing Vph1-GFP. Error bars indicate SD. Bars, 5 µm.

Finally, we investigated the impact of *ldo45Δ* and *ldo16Δ* mutations in LDs. In *ldo45Δ* cells, which express normal Ldo16 levels, LDs were indistinguishable from the controls at all growth stages. Interestingly, in *ldo45Δ ldo16Δ* (or *ldo16Δ*) cells, LDs were normal during the exponential phase but appeared enlarged during early stationary phase (Figs. 5 A and S3 C). Mirroring LD morphology, neutral lipid levels in *ldo45Δ ldo16Δ* mutants are similar to WT and *ldo45Δ* at the exponential phase; however, the double mutant specifically accumulated TAG during early and late stationary phase, whereas SE levels remained constant (Fig. 5 B). Thus, the function of Ldo proteins becomes critical during stationary phase.

Transition from exponential to stationary phases is triggered by nutrient deprivation and involves dramatic rewiring of cellular metabolism, particularly at the level of lipid biosynthesis and turnover (Casanovas et al., 2015). Among the changes, there is a sudden rise in storage lipids (Wang et al., 2014a; Barbosa et al., 2015; Casanovas et al., 2015). During stationary phase, these become an important source of energy through lipophagy, a specific form of LD microautophagy (Singh and Cuervo, 2012; Wang, 2015). Preceding lipophagy, LDs relocalize to ER regions proximal to the vacuole from where they translocate and eventually become degraded within the vacuolar lumen during stationary phase (Wang et al., 2014b). Lipophagy is a slow process peaking 4–6 d after entry in stationary phase and requires macroautophagy components such as *ATG1* (Wang et al., 2014b). In cells lacking Ldo proteins or the seipin component Ldb16, LDs relocalized normally to vacuole proximal regions; however, their translocation into the lumen was strongly impaired (Fig. 5, C and D). In contrast, lipophagy proceeded normally in *ldo45Δ* cells, which express Ldo16, suggesting that this isoform is sufficient for lipophagy. Consistently, in *ldo45Δ ldo16Δ* cells, lipophagy was largely rescued by reexpression of Ldo16 but not by Ldo45 (Figs. 5 E and S3 D). Ldo16 requirement for lipophagy is in agreement with its expression in stationary phase. Moreover, lipophagy impairment in *ldo45Δ ldo16Δ* mutant likely causes TAG accumulation, specifically in stationary phase.

Changes in lipid composition during nutrient deprivation drive vacuolar lipid phase segregation into liquid-ordered (Lo) and disordered domains. These gradually evolve to form a reticulate pattern at late stationary phase (Toulmay and Prinz, 2013). During lipophagy, LDs translocate specifically to Lo domains, highlighting the tight control of this process. In fact, the maintenance of sterol-rich Lo domains appears to require lipophagy (Wang et al., 2014b). Thus, we tested whether the lipophagy defect in *ldo45Δ ldo16Δ* mutants could be explained by abnormal vacuolar domain formation. Indeed, the formation of typical reticulate lipid domains observed at late stationary phase was impaired in *ldo45Δ ldo16Δ* cells (Fig. 5 F). Interestingly, a similar defect was detected in cells lacking Ldb16, which were also defective in lipophagy (Fig. 5 C). How LDs translocate from the ER to the vacuolar Lo domains is not well defined but may involve additional organelle interfaces and signaling events (Elbaz-Alon et al., 2015; Gatta et al., 2015; Moldavski et al., 2015; Murley et al., 2015, 2017). Thus, it will be interesting to temporally and functionally define the contribution of seipin and Ldo16 in this process.

In summary, our study identified Ldo16 and Ldo45 as regulatory subunits of the seipin complex with opposite effects on LDs. Ldo45 appears to modulate TAG storage by reducing Dga1 LD localization and promote LD targeting of some proteins, including Pdr16. Given the roles of seipin in controlling the LD proteome, future studies should dissect the distinct effects of Ldo45 on LD protein targeting. In contrast, Ldo16 does not affect LD proteome and functions primarily upon nutrient depletion, facilitating LD consumption by lipophagy. Remarkably, the relative abundance of these Ldo isoforms is coupled to cellular metabolism. Thus, our data are consistent with a model in which the seipin complex at ER–LD contacts is fine-tuned according to nutrient availability to promote either LD proliferation or consumption.

## Materials and methods

### Reagents

The LD dyes BODIPY^493/503^ (Invitrogen) and MDH (Abgent) were used at 1 µg/ml and 0.1 mM, respectively. Anti-HA (rat monoclonal clone 3F10; 11867431001) antibody was purchased from Roche, anti-Pgk1 (mouse monoclonal clone 22C5D8; 459250) was from Thermo Fisher Scientific, anti-GFP (rat monoclonal clone 3H9) was from ChromoTek, anti-Dpm1 (mouse monoclonal clone 5C5; A6429) was from Thermo Fisher Scientific, and anti-Kar2 (rabbit polyclonal y-115; sc-33630) was from Santa Cruz Biotechnology, Inc. Polyclonal anti-Usa1 (rabbit), anti-Fld1 (rabbit), anti-Ldb16 (rabbit), anti-Erg6 (rabbit), anti-Dga1 (rabbit), and anti-Lro1 (rabbit) antibodies were previously described (Carvalho et al., 2006; Grippa et al., 2015). Polyclonal rabbit anti-Ldo proteins antibody was raised against amino acids 74–87 and 132–146 from Ldo16. For loading controls, anti-Kar2 (Fig. 2 C), anti-Usa1 (Fig. S1, A and B; Fig. S2, C and D; and Fig. S3 A), and anti-Pgk1 (Figs. 3 A and 4 C) were used.

### Yeast strains and plasmids

Protein tagging, promoter replacements, and individual gene deletions were performed by standard PCR-based homologous recombination (Longtine et al., 1998; Janke et al., 2004). Strains with multiple gene deletions were made either by PCR-based homologous recombination or by crossing haploid cells of opposite mating types followed by sporulation and tetrad dissection using standard protocols (Guthrie and Fink, 1991). The strains used are isogenic either to BY4741 (*MATa ura3Δ0 his3Δ1 leu2Δ0 met15Δ0*) or to BY4742 (*MATα his3Δ1 leu2Δ0 lys2Δ0 ura3Δ0*), and primers are listed in Tables S1 and S2, respectively. Plasmids expressing the different combinations of Ldo proteins were gifts from J. Vilardell (Molecular Biology Institute of Barcelona, Barcelona, Spain).

### Media and growth conditions

Cells were grown in yeast extract peptone dextrose (YPD) media (1% yeast extract, 2% bactopeptone, and 2% dextrose), YPGal (1% yeast extract, 2% bactopeptone, and 2% galactose), synthetic complete (SC) media (0.17% yeast nitrogen base, 5 g/liter ammonium sulfate, 2% glucose, and amino acids), SCGal (0.17% yeast nitrogen base, 5 g/liter ammonium sulfate, 2% galactose, and amino acids), or synthetic dropout media supplemented with the corresponding amino acids for plasmid selection. Cells were grown at 30°C and assayed in exponential growth phase at an optical density at λ = 600 nm (OD_600_) between 0.5–1.2 or in early stationary phase at 4–6. For expression of galactose-inducible genes, cells were derepressed for 12–20 h at 30°C in media containing 2% raffinose as the sole carbon source and subsequently diluted into the corresponding media containing 2% galactose for 16–22 h. For lipophagy assays, cells were grown overnight in SC media and then diluted to the log phase (OD = 0.6) culture starting from OD = 0.15. The days 1, 4, and 6 (D1, D4, and D6) are defined as when the same cultures of log phase were grown at 30°C for additional 24, 96, and 144 h, respectively.

### Immunoprecipitation experiments

Large-scale purification of TAP-tagged proteins for mass spectrometry analysis was performed as described previously (Carvalho et al., 2006). Small-scale immunoprecipitations were performed as described previously (Carvalho et al., 2010). In brief, exponential yeast cultures (75–100 OD_600_) were washed and resuspended in 1.4 ml lysis buffer (LB; 50 mM Tris, pH 7.4, 150 mM NaCl, 2 mM MgCl_2_, and cOmplete protease inhibitor [Roche]). Cells were lysed with glass beads, and lysates were cleared by low-speed centrifugation at 4°C. Membranes were pelleted at 50,000 rpm for 25 min at 4°C in an Optima Max Tabletop Ultracentrifuge in a TLA 100.3 rotor (Beckman Coulter). The crude membrane fraction was resuspended in 600 µl LB. 700 µl of LB supplemented with 2% of detergent (digitonin, NP-40, or decyl maltose neopentyl glycol) were added, and membranes were solubilized 2–3 h on a rotating wheel at 4°C. Solubilized membranes were cleared for 15 min at 4°C at full speed in a tabletop centrifuge, and 1.1 ml was used for the immunoprecipitation. TAP-, HA-, and FLAG-tagged proteins were affinity isolated by overnight incubation with calmodulin sepharose 4B beads (17052901; GE Healthcare), anti-HA magnetic beads (88836; Thermo Fisher Scientific), and anti-FLAG M2 magnetic beads (M8823; Sigma-Aldrich), respectively. Eluted proteins were analyzed by SDS-PAGE and immunoblotting. In all experiments, the input lane corresponds with 5% of the total extract used for immunoprecipitation.

### Fluorescence microscopy

Fluorescence microscopy was performed at room temperature (∼23°C) in a Cell Observer high speed microscope (ZEISS) equipped with a CMOS camera (ORCA-Flash 4.0; Hamamatsu Photonics) controlled by Slidebook 6.0 software (3i). A 100× 1.4 NA Plan Apochromat oil immersion objective was used. GFP, BODIPY^493/503^, mCherry, and MDH signals were detected using GFP, RFP, and DAPI filters, respectively, with standard settings. Cells were washed twice with PBS, pH 7.4, before visualization. All quantifications were performed from at least three independent experiments, and in each experiment, ≥100 cells/condition were scored. Data distribution was assumed to be normal, but this was not formally tested. Images were cropped, and contrast and intensity were adjusted with Photoshop CC and grouped with Illustrator CC (2015; Adobe).

For lipophagy quantification, the vacuole border was determined by the Vph1-tdTomato signal (Fig. 5 D) or the differential interference contrast signal (Fig. 5 E) of the corresponding position in the z axis. To image and quantify the vacuolar domains (Fig. 5 F), images were processed by deconvolution and maximal projection using Slidebook 6.0 software.

### LD isolation

LD purification was performed as previously described (Grippa et al., 2015). In brief, cells were grown in 500 ml YPD until stationary phase. Approximately 3,000 OD of cells were centrifuged at 3,000 *g* for 5 min (J J26-XP centrifuge and JLA8100 rotor; Beckman Coulter), washed in milliQ water, preincubated in 0.1 M Tris-HCl, pH 9.5, and 10 mM DTT for 10 min at 30°C, washed, and resuspended in 50 ml spheroplasting buffer (1.2 M sorbitol and 50 mM Tris, pH 7.4). For spheroplast preparation, Zymolyase 20T (Seikagaku Biobusiness) was added (10 µg/OD_600_ U cells) followed by incubation in a water bath at 30°C until a 10-fold drop in OD_600_ was observed (45–60 min). Spheroplasts were recovered by centrifugation (1,000 *g* at 4°C), washed with spheroplasting buffer, and resuspended in breaking buffer (BB: 10 mM MES, Tris, pH 6.9, 12% [wt/wt] Ficoll400, and 0.2 mM EDTA) at a final concentration of 0.3 g of cells (wet weight)/ml, and PMSF (1 mM) and cOmplete were added before homogenization (loose-fitting pestle; 40 strokes) in a Dounce homogenizer on ice. The homogenate was centrifuged (5,000 *g* for 5 min) using rotor JS13.1. The resulting supernatant was transferred into 38-ml Ultra-Clear centrifuge tubes (Beckman Coulter) and adjusted to 19 ml (cell lysate fraction), overlaid with an equal volume of BB, and centrifuged for 45 min at 30,000 rpm in an optima l-100K centrifuge (Beckman Coulter) with an SW-32 swinging bucket rotor. The floating layer was collected from the top of the gradient, and the LDs were further purified. The pellet was collected, resuspended in PBS, pH 7.4, with help of a Dounce homogenizer, and fully dissolved with SDS sample buffer (membranes fraction). The floating layer was gently resuspended in BB (five strokes in a Dounce homogenizer with a loose-fitting pestle), adjusted to 19 ml with BB, transferred to a 38-ml Ultra-Clear tube, and overlaid with 19 ml 10 mM MES-Tris, pH 6.9, 8% (wt/wt) Ficoll 400, and 0.2 mM EDTA. Centrifugation was repeated as before (30,000 rpm for 45 min). The floating layer was collected and resuspended in 19 ml 10 mM MES-Tris, pH 6.9, 0.6 M sorbitol, 8% (wt/wt) Ficoll 400, and 0.2 mM EDTA, transferred to 38-ml Ultra-Clear tubes, overlaid with 19 ml 10 mM MES-Tris, pH 6.9, 0.25 M sorbitol, and 0.2 mM EDTA, and then centrifuged once more for 30 min at 30,000 rpm. The recovered high-purity top LD fraction was snap frozen, stored at −80°C, and used subsequently for proteomics.

### Protein mass spectrometry

TCA-precipitated proteins were resuspended in 6 M urea and 200 mM ammonium bicarbonate before reduction (10 mM DTT) and alkylation (20 mM iodoacetamide). Samples were diluted to 2 M urea and digested with trypsin (1:10 wt/wt) overnight at 37°C. Tryptic peptide mixtures were desalted using a C18 UltraMicroSpin column using three washes with 0.1% formic acid in water followed by an elution step with 0.1% formic acid in a 1:1 mix of water and acetonitrile (Rappsilber et al., 2007).

Samples were analyzed in a LTQ-Orbitrap Velos Pro mass spectrometer (Thermo Fisher Scientific) coupled to nano-LC (Proxeon) equipped with a reverse-phase chromatography 12-cm column with an inner diameter of 75 µm, packed with 5-µm C18 particles (Nikkyo Technos Co., Ltd.). Chromatographic gradients were set from 93% buffer A, 7% buffer B to 65% buffer A, or 35% buffer B in 60 min with a flow rate of 300 nl/min. Buffer A contained 0.1% formic acid in water, and buffer B contained 0.1% formic acid in acetonitrile. The instrument was operated in data-dependent acquisition mode and full mass spectrometry scans, and 1-µm scans at a resolution of 60,000 were used over a mass range of m/z 250–2,000 with detection in the Orbitrap. After each survey, scans of the top 20 most intense ions with multiple charged ions above a threshold ion count of 5,000 were selected for fragmentation at a normalized collision energy of 35%. Fragment ion spectra produced via collision-induced dissociation were acquired in the linear ion trap. All data were acquired with Xcalibur software (v2.2; Thermo Fisher Scientific).

Acquired data were analyzed using the Proteome Discoverer software suite (v1.3.0.339; Thermo Fisher Scientific), and the MASCOT search engine (v2.3; Matrix Science) was used for peptide identification. Data were searched against a database containing all yeast proteins according to the Saccharomyces Genome Database (Cherry et al., 2012) plus the most common contaminants (Bunkenborg et al., 2010). A precursor ion mass tolerance of 7 ppm at the MS1 level was used, and up to three miscleavages for trypsin were allowed. The fragment ion mass tolerance was set to 0.5 D. Oxidation of methionine and protein acetylation at the N terminus were defined as variable modifications. Carbamidomethylation on cysteines was set as a fixed modification. The identified peptides were filtered using a false discovery rate of <5%.

Protein areas were normalized intra- and intersamples by the median of the Log area. A linear modeling approach implemented in *lmFit* function and the empirical Bayes statistics implemented in *eBayes* and *topTable* functions of the Bioconductor *limma* package were used to perform a differential protein abundance analysis (Gentleman et al., 2004; Smyth, 2004). The normalized protein areas of different yeast mutants were compared with WT samples. Protein p-values were calculated with *limma* and were adjusted with Benjamini–Hochberg method (Benjamini and Hochberg, 1995). A value of 0.05 was used as cutoff.

### Yeast lipid extraction

Yeast cell pellets (∼10 OD_U_) were resuspended in 1 ml of 155 mM ammonium formate and lysed at 4°C with 400 µl of acid-washed glass beads using a cell disruptor (3 × 1 min). Lysates corresponding with 0.4 OD_U_ per 200 µl were spiked with 30 µl of internal standard mixture containing 250 pmol cholesterol ester (10:0), 60 pmol cerimide (18:1;2/17:0;1), 60 pmol diacylglycerol (17:0/17:0 + [2]H5), 75 pmol phosphatidylcholine (16:0/16:0 + [2]H6), 110 pmol internal standard phosphatidylethanolamine (15:0/18:1 + [2]H7), 65 pmol phosphatidylinositol (15:0/18:1 + [2]H7), and 35 pmol TAG (17:0/17:1/17:0 + [2]H5). The samples were subsequently extracted with 990 µl chloroform/methanol (2:1; vol/vol) for 2 h (1,400 rpm at 4°C) using a ThermoMixer (Eppendorf). The lower organic phase was collected after centrifugation (3,000 *g* for 2 min at 4°C) and vacuum evaporated. Finally, the lipid extracts were reconstituted in 100 µl chloroform/methanol (1:2; vol/vol) before their analysis by mass spectrometry.

### Mass spectrometric lipid analysis

Lipid extracts were analyzed by MS^ALL^ using an Orbitrap Fusion Tribrid (Thermo Fisher Scientific) equipped with a robotic nanoflow ion source, TriVersa NanoMate (Advion Biosciences). In positive ion mode, aliquots of 2:1 lipid extracts were infused in chloroform/methanol/2-propanol (1:2:4; vol/vol/vol) with 7.5 mM ammonium formate using a back pressure of 1.25 psi and an ionization voltage of +0.96 kV. MS^ALL^ analysis was performed using high-resolution Fourier transform mass analyzer (FTMS) analysis of the m/z range 500–1,400 and sequential FTMS2 analysis of all precursor ions in the m/z range of 400–1,050 (Almeida et al., 2015).

### EM

Cells were cryoimmobilized by high-pressure freezing using an EM HPM100 microscope (Leica Microsystems). Freeze substitution of frozen samples was performed in an automatic freeze substitution system (EM AFS-2; Leica Microsystems) using acetone containing 0.1% of uranyl acetate and 1% water for 3 d at −90°C. On the fourth day, the temperature was slowly increased by 5°C/h to −45°C. At this temperature, samples were rinsed in acetone and subsequently infiltrated and embedded in Lowicryl HM20 for 3 d. 90-nm sections were obtained using a diamond knife (Diatome) on a UC7 ultramicrotome (Leica Microsystems), transferred to formvar-coated 100 mesh Cu grids, and then poststained for 5 min with 2% uranyl acetate and 10 min with Reynold’s lead citrate. Grids were imaged using a Tecnai 12 transmission electron microscope (FEI) operated at 120 kV with a OneView digital camera (Gatan).

### Online supplemental material

Fig. S1 shows two Ldo isoforms interacting with the seipin complex and localizing to LDs. Fig. S2 shows how Ldo45 is required for LD recruitment of Pdr16. Fig. S3 shows how Ldo proteins are required for normal LD morphology. Table S1 shows yeast strains used in this study. Table S2 shows primers used in this study.

## Supplementary Material

Supplemental Materials

## References

[bib1] Almeida R., Pauling J.K., Sokol E., Hannibal-Bach H.K., and Ejsing C.S. 2015 Comprehensive lipidome analysis by shotgun lipidomics on a hybrid quadrupole-orbitrap-linear ion trap mass spectrometer. J. Am. Soc. Mass Spectrom. 26:133–148. 10.1007/s13361-014-1013-x 25391725

[bib2] Athenstaedt K., and Daum G. 2003 YMR313c/TGL3 encodes a novel triacylglycerol lipase located in lipid particles of Saccharomyces cerevisiae. J. Biol. Chem. 278:23317–23323. 10.1074/jbc.M302577200 12682047

[bib3] Barbosa A.D., Sembongi H., Su W.M., Abreu S., Reggiori F., Carman G.M., and Siniossoglou S. 2015 Lipid partitioning at the nuclear envelope controls membrane biogenesis. Mol. Biol. Cell. 26:3641–3657. 10.1091/mbc.E15-03-0173 26269581 PMC4603934

[bib4] Benjamini Y., and Hochberg Y. 1995 Controlling the false discovery rate: a practical and powerful approach to multiple testing. J. R. Stat. Soc. B. 57:289–300.

[bib5] Binns D., Lee S., Hilton C.L., Jiang Q.-X., and Goodman J.M. 2010 Seipin is a discrete homooligomer. Biochemistry. 49:10747–10755. 10.1021/bi1013003 21062080 PMC3086013

[bib6] Bunkenborg J., García G.E., Paz M.I.P., Andersen J.S., and Molina H. 2010 The minotaur proteome: avoiding cross-species identifications deriving from bovine serum in cell culture models. Proteomics. 10:3040–3044. 10.1002/pmic.201000103 20641139

[bib7] Cartwright B.R., Binns D.D., Hilton C.L., Han S., Gao Q., and Goodman J.M. 2014 Seipin performs dissectible functions in promoting lipid droplet biogenesis and regulating droplet morphology. Mol. Biol. Cell. 26:726–739. 10.1091/mbcE14-08-1303 25540432 PMC4325842

[bib8] Carvalho P., Goder V., and Rapoport T.A. 2006 Distinct ubiquitin-ligase complexes define convergent pathways for the degradation of ER proteins. Cell. 126:361–373. 10.1016/j.cell.2006.05.043 16873066

[bib9] Carvalho P., Stanley A.M., and Rapoport T.A. 2010 Retrotranslocation of a misfolded luminal ER protein by the ubiquitin-ligase Hrd1p. Cell. 143:579–591. 10.1016/j.cell.2010.10.028 21074049 PMC3026631

[bib10] Casanovas A., Sprenger R.R., Tarasov K., Ruckerbauer D.E., Hannibal-Bach H.K., Zanghellini J., Jensen O.N., and Ejsing C.S. 2015 Quantitative analysis of proteome and lipidome dynamics reveals functional regulation of global lipid metabolism. Chem. Biol. 22:412–425. 10.1016/j.chembiol.2015.02.007 25794437

[bib11] Cherry J.M., Hong E.L., Amundsen C., Balakrishnan R., Binkley G., Chan E.T., Christie K.R., Costanzo M.C., Dwight S.S., Engel S.R., 2012. Saccharomyces Genome Database: the genomics resource of budding yeast. Nucleic Acids Res. Jan;40(Database issue):D700-5. 10.1093/nar/gkr1029 PMC3245034 22110037

[bib12] Dubots E., Cottier S., Péli-Gulli M.P., Jaquenoud M., Bontron S., Schneiter R., and De Virgilio C. 2014 TORC1 regulates Pah1 phosphatidate phosphatase activity via the Nem1/Spo7 protein phosphatase complex. PLoS One. 9:e104194 10.1371/journal.pone.0104194 25117580 PMC4130541

[bib13] Eisenberg-Bord M., Mari M., Weill U., Rosenfeld-Gur E., Moldavski O., Castro I.G., Soni K.G., Harpaz N., Levine T.P., Futerman A.H., 2018 Identification of seipin-linked factors that act as determinants of a lipid droplet subpopulation. J. Cell Biol. 10.1083/jcb.201704122 PMC5748981 29187527

[bib14] Elbaz-Alon Y., Eisenberg-Bord M., Shinder V., Stiller S.B., Shimoni E., Wiedemann N., Geiger T., and Schuldiner M. 2015 Lam6 Regulates the Extent of Contacts between Organelles. Cell Reports. 12:7–14. 10.1016/j.celrep.2015.06.022 26119743 PMC4518459

[bib15] Fei W., Shui G., Gaeta B., Du X., Kuerschner L., Li P., Brown A.J., Wenk M.R., Parton R.G., and Yang H. 2008 Fld1p, a functional homologue of human seipin, regulates the size of lipid droplets in yeast. J. Cell Biol. 180:473–482. 10.1083/jcb.200711136 18250201 PMC2234226

[bib16] Fei W., Shui G., Zhang Y., Krahmer N., Ferguson C., Kapterian T.S., Lin R.C., Dawes I.W., Brown A.J., Li P., 2011 A role for phosphatidic acid in the formation of “supersized” lipid droplets. PLoS Genet. 7:e1002201 10.1371/journal.pgen.1002201 21829381 PMC3145623

[bib17] Gatta A.T., Wong L.H., Sere Y.Y., Calderón-Noreña D.M., Cockcroft S., Menon A.K., and Levine T.P. 2015 A new family of StART domain proteins at membrane contact sites has a role in ER-PM sterol transport. eLife. 4:1–21. 10.7554/eLife.07253 PMC4463742 26001273

[bib18] Gentleman R.C., Carey V.J., Bates D.M., Bolstad B., Dettling M., Dudoit S., Ellis B., Gautier L., Ge Y., Gentry J., 2004 Bioconductor: open software development for computational biology and bioinformatics. Genome Biol. 5:R80 10.1186/gb-2004-5-10-r80 15461798 PMC545600

[bib19] Grillitsch K., Connerth M., Köfeler H., Arrey T.N., Rietschel B., Wagner B., Karas M., and Daum G. 2011 Lipid particles/droplets of the yeast Saccharomyces cerevisiae revisited: lipidome meets proteome. Biochim. Biophys. Acta. 1811:1165–1176. 10.1016/j.bbalip.2011.07.015 21820081 PMC3229976

[bib20] Grippa A., Buxó L., Mora G., Funaya C., Idrissi F.Z., Mancuso F., Gomez R., Muntanyà J., Sabidó E., and Carvalho P. 2015 The seipin complex Fld1/Ldb16 stabilizes ER-lipid droplet contact sites. J. Cell Biol. 211:829–844. 10.1083/jcb.201502070 26572621 PMC4657162

[bib21] Guthrie C., and Fink G.R. 1991 Guide to Yeast Genetics and Molecular Biology. 194. 1-863 pp. 2005781

[bib22] Han S., Binns D.D., Chang Y.-F., and Goodman J.M. 2015 Dissecting seipin function: the localized accumulation of phosphatidic acid at ER/LD junctions in the absence of seipin is suppressed by Sei1p(ΔNterm) only in combination with Ldb16p. BMC Cell Biol. 16:29 10.1186/s12860-015-0075-3 26637296 PMC4670494

[bib23] Holič R., Simova Z., Ashlin T., Pevala V., Poloncova K., Tahotna D., Kutejova E., Cockcroft S., and Griac P. 2014 Phosphatidylinositol binding of Saccharomyces cerevisiae Pdr16p represents an essential feature of this lipid transfer protein to provide protection against azole antifungals. Biochim. Biophys. Acta - Mol. Cell Biol. Lipids. 1841:1483–1490. 10.1016/j.bbalip.2014.07.014 PMC4331669 25066473

[bib24] Jacquier N., Choudhary V., Mari M., Toulmay A., Reggiori F., and Schneiter R. 2011 Lipid droplets are functionally connected to the endoplasmic reticulum in Saccharomyces cerevisiae. J. Cell Sci. 124:2424–2437. 10.1242/jcs.076836 21693588

[bib25] Janke C., Magiera M.M., Rathfelder N., Taxis C., Reber S., Maekawa H., Moreno-Borchart A., Doenges G., Schwob E., Schiebel E., and Knop M. 2004 A versatile toolbox for PCR-based tagging of yeast genes: new fluorescent proteins, more markers and promoter substitution cassettes. Yeast. 21:947–962. 10.1002/yea.1142 15334558

[bib26] Kory N., Thiam A.-R., Farese R.V. Jr., and Walther T.C. 2015 Protein Crowding Is a Determinant of Lipid Droplet Protein Composition. Dev. Cell. 34:351–363. 10.1016/j.devcel.2015.06.007 26212136 PMC4536137

[bib27] Kory N., Farese R.V. Jr., and Walther T.C. 2016 Targeting Fat: Mechanisms of Protein Localization to Lipid Droplets. Trends Cell Biol. 26:535–546. 10.1016/j.tcb.2016.02.007 26995697 PMC4976449

[bib28] Krahmer N., Farese R.V. Jr., and Walther T.C. 2013 Balancing the fat: lipid droplets and human disease. EMBO Mol. Med. 5:973–983. 10.1002/emmm.201100671 23740690 PMC3721468

[bib29] Longtine M.S., McKenzie A. III, Demarini D.J., Shah N.G., Wach A., Brachat A., Philippsen P., and Pringle J.R. 1998 Additional modules for versatile and economical PCR-based gene deletion and modification in Saccharomyces cerevisiae. Yeast. 14:953–961. 10.1002/(SICI)1097-0061(199807)14:10<953::AID-YEA293>3.0.CO;2-U 9717241

[bib30] Markgraf D.F., Klemm R.W., Junker M., Hannibal-Bach H.K., Ejsing C.S., and Rapoport T.A. 2014 An ER protein functionally couples neutral lipid metabolism on lipid droplets to membrane lipid synthesis in the ER. Cell Reports. 6:44–55. 10.1016/j.celrep.2013.11.046 24373967 PMC3947819

[bib31] Miura F., Kawaguchi N., Sese J., Toyoda A., Hattori M., Morishita S., and Ito T. 2006 A large-scale full-length cDNA analysis to explore the budding yeast transcriptome. Proc. Natl. Acad. Sci. USA. 103:17846–17851. 10.1073/pnas.0605645103 17101987 PMC1693835

[bib32] Moldavski O., Amen T., Levin-Zaidman S., Eisenstein M., Rogachev I., Brandis A., Kaganovich D., and Schuldiner M. 2015 Lipid Droplets Are Essential for Efficient Clearance of Cytosolic Inclusion Bodies. Dev. Cell. 33:603–610. 10.1016/j.devcel.2015.04.015 26004510

[bib33] Murley A., Sarsam R.D., Toulmay A., Yamada J., Prinz W.A., and Nunnari J. 2015 Ltc1 is an ER-localized sterol transporter and a component of ER-mitochondria and ER-vacuole contacts. J. Cell Biol. 209:539 10.1083/jcb.201502033 25987606 PMC4442815

[bib34] Murley A., Yamada J., Niles B.J., Toulmay A., Prinz W.A., and Powers T. 2017 Sterol transporters at membrane contact sites regulate TORC1 and TORC2 signaling. J. Cell Biol. 216:1–11. 10.1083/jcb.201610032 PMC5584152 28774891

[bib35] Oelkers P., Tinkelenberg A., Erdeniz N., Cromley D., Billheimer J.T., and Sturley S.L. 2000 A lecithin cholesterol acyltransferase-like gene mediates diacylglycerol esterification in yeast. J. Biol. Chem. 275:15609–15612. 10.1074/jbc.C000144200 10747858

[bib36] Oelkers P., Cromley D., Padamsee M., Billheimer J.T., and Sturley S.L. 2002 The DGA1 gene determines a second triglyceride synthetic pathway in yeast. J. Biol. Chem. 277:8877–8881. 10.1074/jbc.M111646200 11751875

[bib37] Pagac M., Cooper D.E., Qi Y., Lukmantara I.E., Mak H.Y., Wu Z., Tian Y., Liu Z., Lei M., Du X., 2016 SEIPIN Regulates Lipid Droplet Expansion and Adipocyte Development by Modulating the Activity of Glycerol-3-phosphate Acyltransferase. Cell Reports. 17:1546–1559. 10.1016/j.celrep.2016.10.037 27806294 PMC5647143

[bib38] Pol A., Gross S.P., and Parton R.G. 2014 Biogenesis of the multifunctional lipid droplet: lipids, proteins, and sites. J. Cell Biol. 204:635–646. 10.1083/jcb.201311051 24590170 PMC3941045

[bib39] Rappsilber J., Mann M., and Ishihama Y. 2007 Protocol for micro-purification, enrichment, pre-fractionation and storage of peptides for proteomics using StageTips. Nat. Protoc. 2:1896–1906. 10.1038/nprot.2007.261 17703201

[bib40] Ren J., Pei-Chen Lin C., Pathak M.C., Temple B.R.S., Nile A.H., Mousley C.J., Duncan M.C., Eckert D.M., Leiker T.J., Ivanova P.T., 2014 A Phosphatidylinositol Transfer Protein Integrates Phosphoinositide Signaling with Lipid Droplet Metabolism to Regulate a Developmental Program of Nutrient Stress-Induced Membrane Biogenesis. Mol. Biol. Cell. 25:712–727. 10.1091/mbc.E13-11-0634 24403601 PMC3937096

[bib41] Salo V.T., Belevich I., Li S., Karhinen L., Vihinen H., Vigouroux C., Magré J., Thiele C., Hölttä-Vuori M., Jokitalo E., and Ikonen E. 2016 Seipin regulates ER-lipid droplet contacts and cargo delivery. EMBO J. 35:2699–2716. 10.15252/embj.201695170 27879284 PMC5167346

[bib42] Schreiber K., Csaba G., Haslbeck M., and Zimmer R. 2015 Alternative splicing in next generation sequencing data of saccharomyces cerevisiae. PLoS One. 10:e0140487 10.1371/journal.pone.0140487 26469855 PMC4607428

[bib43] Singh R., and Cuervo A.M. 2012 Lipophagy: connecting autophagy and lipid metabolism. Int. J. Cell Biol. 2012:282041 10.1155/2012/282041 22536247 PMC3320019

[bib44] Smyth G.K. 2004 Linear models and empirical bayes methods for assessing differential expression in microarray experiments. Stat. Appl. Genet. Mol. Biol. 3:e3 10.2202/1544-6115.1027 16646809

[bib45] Szymanski K.M., Binns D., Bartz R., Grishin N.V., Li W.P., Agarwal A.K., Garg A., Anderson R.G., and Goodman J.M. 2007 The lipodystrophy protein seipin is found at endoplasmic reticulum lipid droplet junctions and is important for droplet morphology. Proc. Natl. Acad. Sci. USA. 104:20890–20895. 10.1073/pnas.0704154104 18093937 PMC2409237

[bib46] Toulmay A., and Prinz W.A. 2013 Direct imaging reveals stable, micrometer-scale lipid domains that segregate proteins in live cells. J. Cell Biol. 202:35–44. 10.1083/jcb.201301039 23836928 PMC3704982

[bib47] Walther T.C., and Farese R.V. Jr 2012 Lipid droplets and cellular lipid metabolism. Annu. Rev. Biochem. 81:687–714. 10.1146/annurev-biochem-061009-102430 22524315 PMC3767414

[bib48] Wang C.-W. 2015 Lipid droplets, lipophagy, and beyond. Biochim. Biophys. Acta - Mol. Cell Biol. Lipids. 1861:793–805. 10.1016/j.bbalip.2015.12.010 26713677

[bib49] Wang C.-W., Miao Y.-H., and Chang Y.-S. 2014 a A sterol-enriched vacuolar microdomain mediates stationary phase lipophagy in budding yeast. J. Cell Biol. 206:357–366. 10.1083/jcb.201404115 25070953 PMC4121974

[bib50] Wang C.W., Miao Y.H., and Chang Y.S. 2014 b Control of lipid droplet size in budding yeast requires the collaboration between Fld1 and Ldb16. J. Cell Sci. 127:1214–1228. 10.1242/jcs.137737 24434579

[bib51] Wang H., Becuwe M., Housden B.E., Chitraju C., Porras A.J., Graham M.M., Liu X.N., Thiam A.R., Savage D.B., Agarwal A.K., 2016 Seipin is required for converting nascent to mature lipid droplets. eLife. 5:1–28. 10.7554/eLife.16582 PMC5035145 27564575

[bib52] Wolinski H., Kolb D., Hermann S., Koning R.I., and Kohlwein S.D. 2011 A role for seipin in lipid droplet dynamics and inheritance in yeast. J. Cell Sci. 124:3894–3904. 10.1242/jcs.091454 22100922

[bib53] Wolinski H., Hofbauer H.F., Hellauer K., Cristobal-Sarramian A., Kolb D., Radulovic M., Knittelfelder O.L., Rechberger G.N., and Kohlwein S.D. 2015 Seipin is involved in the regulation of phosphatidic acid metabolism at a subdomain of the nuclear envelope in yeast. Biochim. Biophys. Acta - Mol. Cell Biol. Lipids. 1851:1450–1464. 10.1016/j.bbalip.2015.08.003 26275961

[bib54] Yang H., Galea A., Sytnyk V., and Crossley M. 2012 Controlling the size of lipid droplets: lipid and protein factors. Curr. Opin. Cell Biol. 24:509–516. 10.1016/j.ceb.2012.05.012 22726586

