# Supplementary material for: Regulation of lipid droplets by metabolically controlled Ldo isoforms
Source: J Cell Biol. 2018 Jan 2;217(1):127–38. doi: 10.1083/jcb.201704115 (PMC5748980; doi:10.1083/jcb.201704115)
Supplement: Supplemental Materials [file JCB_201704115_sm.pdf]

Teixeira et al., https://doi.org/10.1083/jcb.201704115

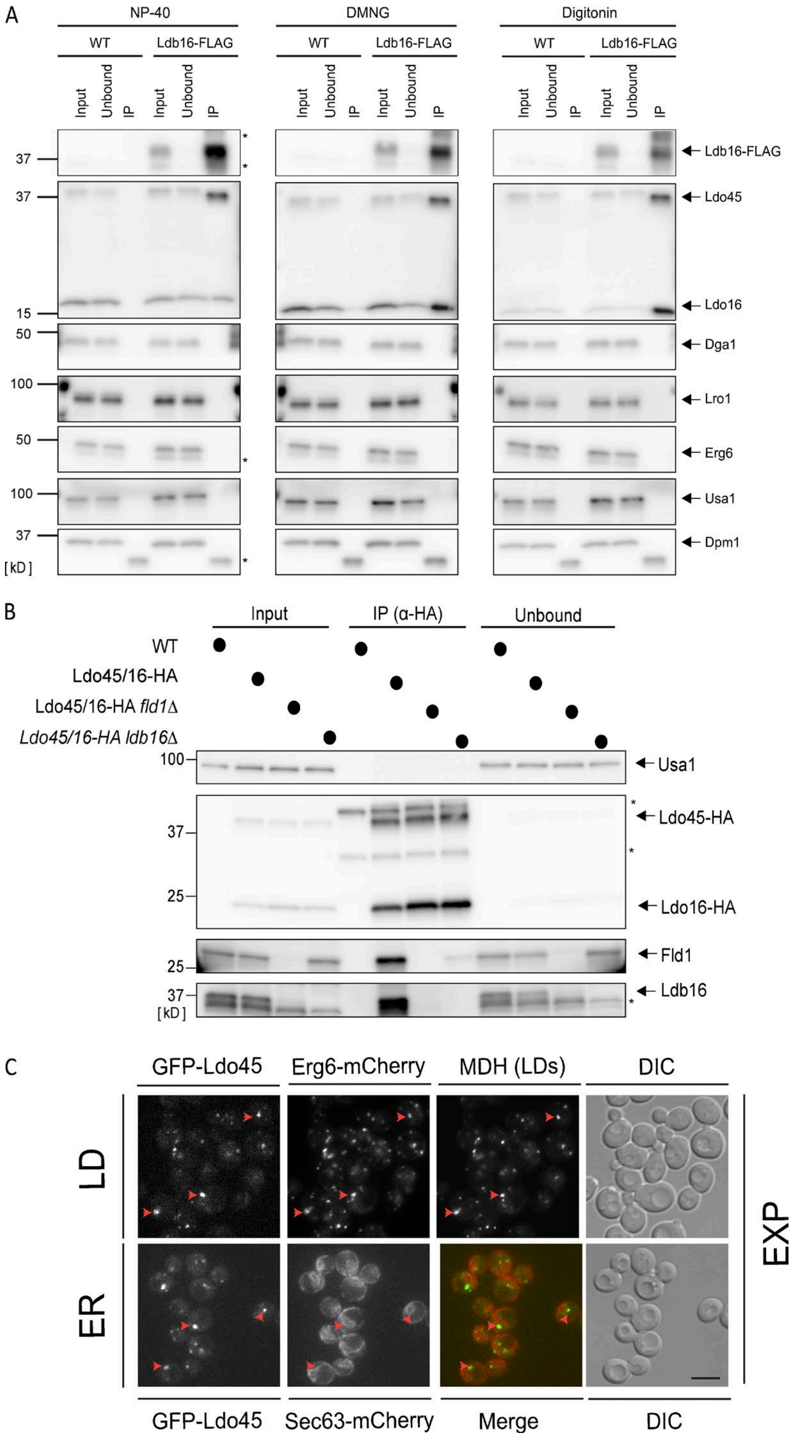

Figure S1. **Two Ldo isoforms interact with the seipin complex and localize to LDs. (A)** Endogenous Ldb16-FLAG coimmunoprecipitates specifically with Ldo proteins under various conditions. Crude membranes were solubilized in the indicated detergents, and solubilized extracts were subjected to immunoprecipitation (IP) with anti-FLAG–coupled magnetic beads. Eluted proteins were analyzed by Western blotting with the indicated antibodies. **(B)** Endogenous Ldb16 and Fld1 coprecipitate with Ldo isoforms. Detergent-solubilized extracts prepared from cells with the indicated genotype were subjected to immunoprecipitation. Eluted proteins were analyzed by Western blotting with antibodies for the indicated proteins. Asterisks indicate nonspecific bands. **(C)** Cells expressing *CYC1*-driven GFP-Ldo45 from the endogenous *LDO45* locus colocalizes with LDs adjacent to the perinuclear ER. Cells were grown in YPD to the exponential (EXP) phase. Endogenously tagged Erg6-mCherry and Sec63-mCherry were used as markers for LDs and the ER, respectively. LDs were also visualized by MDH staining. Red arrowheads indicate colocalization. Bar, 2 µm. DIC, differential interference contrast.

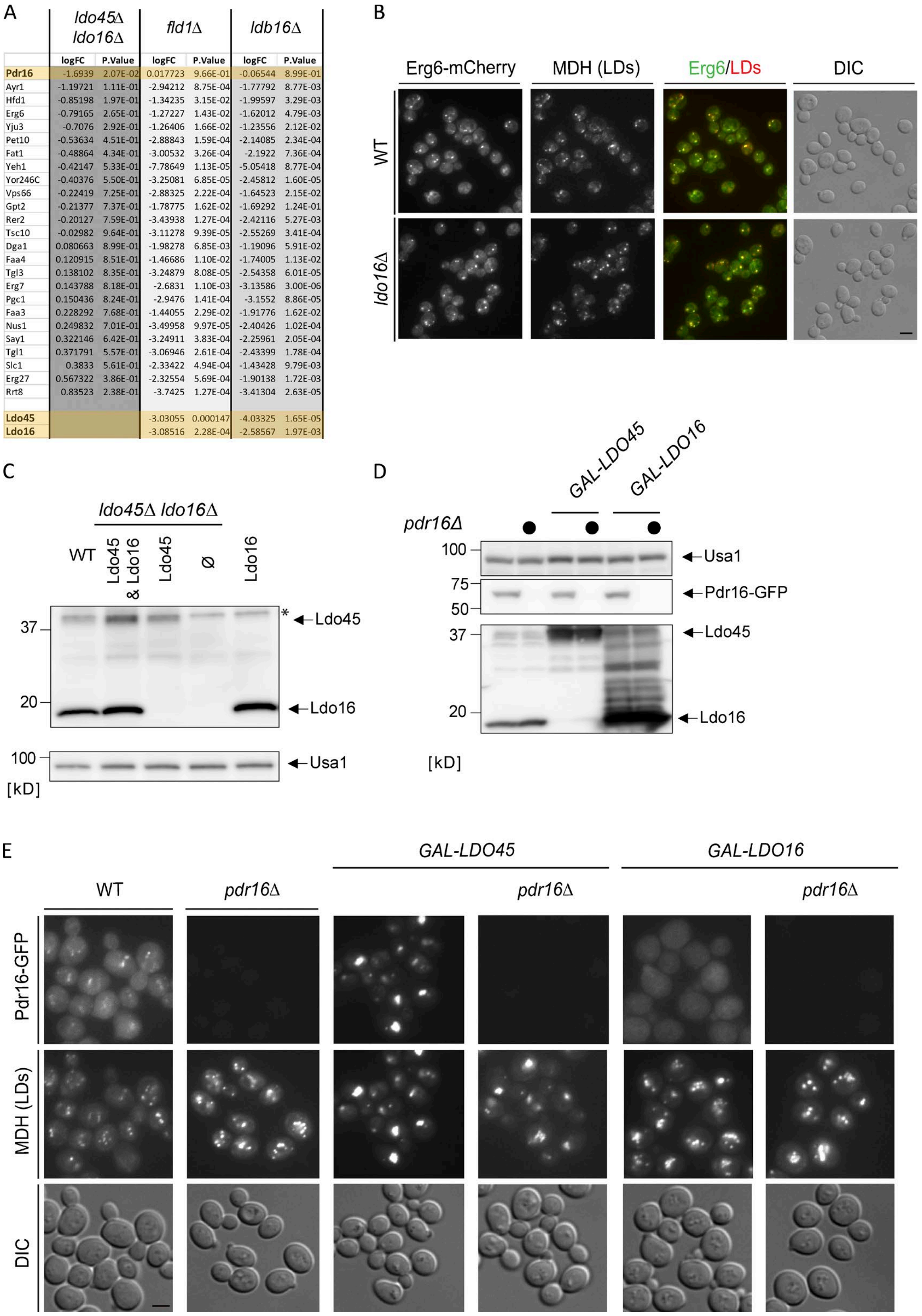

	ldo45Δ ldo16Δ		*fld1Δ*		*ldb16Δ*	
	logFC	P.Value	logFC	P.Value	logFC	P.Value
**Pdr16**	-1.6939	2.07E-02	0.017723	9.66E-01	-0.06544	8.99E-01
Ayr1	-1.19721	1.11E-01	-2.94212	8.75E-04	-1.77792	8.77E-03
Hfd1	-0.85198	1.97E-01	-1.34235	3.15E-02	-1.99597	3.29E-03
Erg6	-0.79165	2.65E-01	-1.27227	1.43E-02	-1.62012	4.79E-03
Yju3	-0.7076	2.92E-01	-1.26406	1.66E-02	-1.23556	2.12E-02
Pet10	-0.53634	4.51E-01	-2.88843	1.59E-04	-2.14085	2.34E-04
Fat1	-0.48864	4.34E-01	-3.00532	3.26E-04	-2.1922	7.36E-04
Yeh1	-0.42147	5.33E-01	-7.78649	1.13E-05	-5.05418	8.77E-04
Yor246C	-0.40376	5.50E-01	-3.25081	6.85E-05	-2.45812	1.60E-05
Vps66	-0.22419	7.25E-01	-2.88325	2.22E-04	-1.64523	2.15E-02
Gpt2	-0.21377	7.37E-01	-1.78775	1.62E-02	-1.69292	1.24E-01
Rer2	-0.20127	7.59E-01	-3.43938	1.27E-04	-2.42116	5.27E-03
Tsc10	-0.02982	9.64E-01	-3.11278	9.39E-05	-2.55269	3.41E-04
Dga1	0.080663	8.99E-01	-1.98278	6.85E-03	-1.19096	5.91E-02
Faa4	0.120915	8.51E-01	-1.46686	1.10E-02	-1.74005	1.13E-02
Tgl3	0.138102	8.35E-01	-3.24879	8.08E-05	-2.54358	6.01E-05
Erg7	0.143788	8.18E-01	-2.6831	1.10E-03	-3.13586	3.00E-06
Pgc1	0.150436	8.24E-01	-2.9476	1.41E-04	-3.1552	8.86E-05
Faa3	0.228292	7.68E-01	-1.44055	2.29E-02	-1.91776	1.62E-02
Nus1	0.249832	7.01E-01	-3.49958	9.97E-05	-2.40426	1.02E-04
Say1	0.322146	6.42E-01	-3.24911	3.83E-04	-2.25961	2.05E-04
Tgl1	0.371791	5.57E-01	-3.06946	2.61E-04	-2.43399	1.78E-04
Slc1	0.3833	5.61E-01	-2.33422	4.94E-04	-1.43428	9.79E-03
Erg27	0.567322	3.86E-01	-2.32554	5.69E-04	-1.90138	1.72E-03
Rrt8	0.83523	2.38E-01	-3.7425	1.27E-04	-3.41304	2.63E-05
**Ldo45**			-3.03055	0.000147	-4.03325	1.65E-05
**Ldo16**			-3.08516	2.28E-04	-2.58567	1.97E-03

Figure S2. **Ldo45 is required for LD recruitment of Pdr16. (A)** Fold change (FC) of proteins in LDs isolated from the indicated mutants in relation to LDs from WT cells as determined by label-free quantitative proteomics. **(B)** Localization of endogenously expressed Erg6-mCherry in cells with the indicated genotype. LDs were visualized with MDH. **(C)** Analysis of expression levels of plasmid-borne Ldo proteins. Crude membrane extracts prepared from WT cells or the *ldo45Δ ldo16Δ* mutant bearing a plasmid expressing the indicated Ldo isoform or isoforms. Ldo proteins detected with anti-Ldo antibody. Usa1 was used as loading control. The asterisk indicates a nonspecific band. **(D)** Levels of Pdr16-GFP and Ldo isoforms in cells with the indicated genotype. Usa1 was used as a loading control. **(E)** Localization of Pdr16-GFP in cells with the indicated genotype. Ldo isoforms were overexpressed from the *GAL* promoter. Cells were grown to exponential phase in rich media supplemented with 2% galactose. LDs were visualized by MDH staining. Bars, 5 µm. DIC, differential interference contrast.

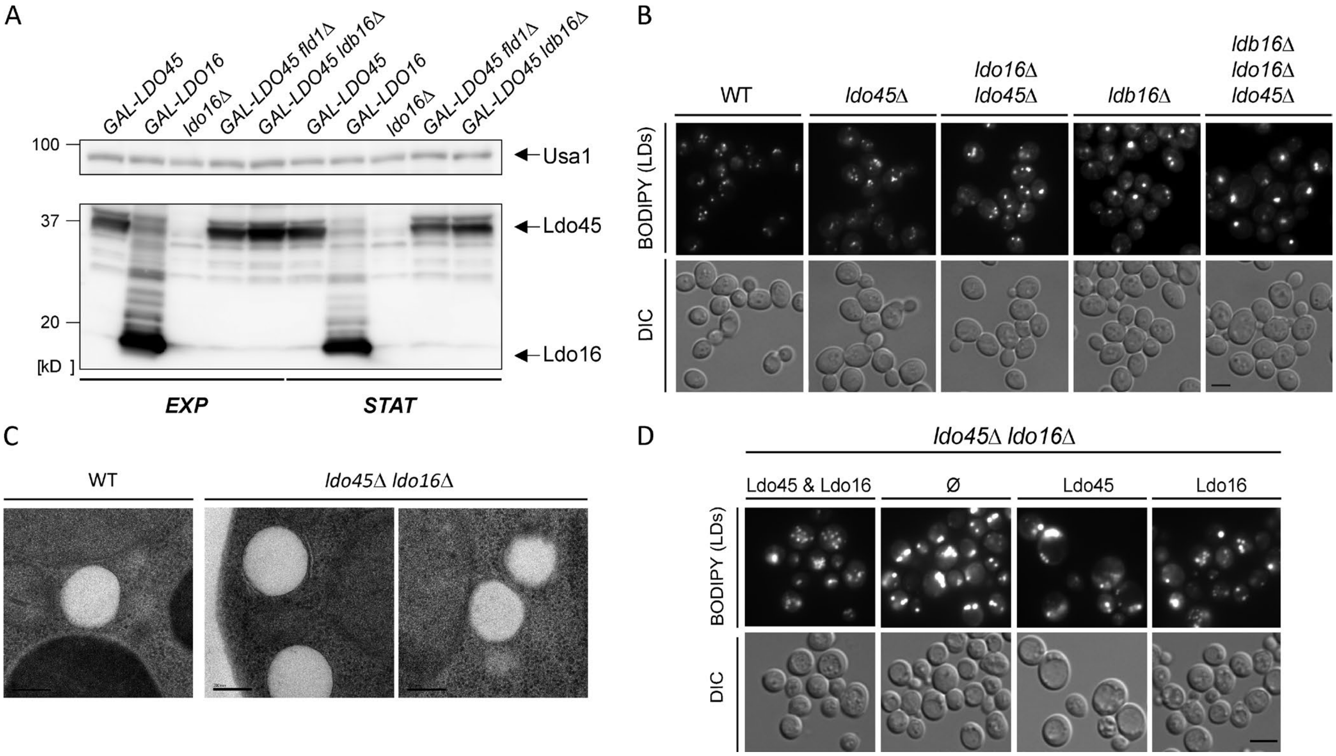

Figure S3. **Ldo proteins are required for normal LD morphology. (A)** Levels of Ldo isoforms in cells with the indicated genotypes both in exponential (EXP) and stationary (STAT) phases. Ldo proteins detected with anti-Ldo antibody. Usa1 was analyzed as loading control. **(B)** Visualization of BODIPY-stained LDs in cells with the indicated genotypes grown in YPGAL to stationary phase. **(C)** Thin-section electron micrographs of WT and *ldo45Δ ldo16Δ* cells grown in YPD media to early stationary phase. **(D)** Representative images of cells grown as indicated in Fig. 5 E. Bars: (B and D) 2 µm; (C) 200 nm.

Table S1. **Yeast strains used in this study**

Strain	Genotype	Origin
yPC1505	*MATa ura3Δ0 his3Δ1 leu2Δ0 met15Δ0*	BY4741
yPC1506	*MATα ura3Δ0 his3Δ1 leu2Δ0 lys2Δ0*	BY4742
yPC3389	*MATa ura3Δ0 his3Δ1 leu2Δ0 met15Δ0 FLD1-TAP-HIS5*	This study
yPC3421	*MATa ura3Δ0 his3Δ1 leu2Δ0 met15Δ0 LDB16-TAP-HIS5*	This study
yPC3541	*MATα ura3Δ0 his3Δ1 leu2Δ0 met15Δ0 ymr147w::KANR*	This study
yPC3543	*MATα ura3Δ0 his3Δ1 leu2Δ0 met15Δ0 ymr148w::KANR*	This study
yPC3546	*MATα ura3Δ0 his3Δ1 leu2Δ0 met15Δ0 ymr147w-148w::KANR*	This study
yPC4060	*MATa ura3Δ0 his3Δ1 leu2Δ0 met15Δ0 dga1::KANR*	KO collection
yPC4069	*MATa ura3Δ0 his3Δ1 leu2Δ0 met15Δ0 pdr16::KANR*	KO collection
yPC4086	*MATa ura3Δ0 his3Δ1 leu2Δ0 met15Δ0 lro1::KANR*	KO collection
yPC4114	*MATa ura3Δ0 his3Δ1 leu2Δ0 met15Δ0 ERG6-mCherry-KANR*	This study
yPC4278	*MATa ura3Δ0 his3Δ1 leu2Δ0 met15Δ0 HIS3-GAL1-LDO16*	This study
yPC4279	*MATa ura3Δ0 his3Δ1 leu2Δ0 met15Δ0 HIS3-GAL1-LDO16 ERG6-mCherry-KANR*	This study
yPC4288	*MATa ura3Δ0 his3Δ1 leu2Δ0 met15Δ0 LDO16-3HA-HIS3*	This study
yPC4289	*MATa ura3Δ0 his3Δ1 leu2Δ0 met15Δ0 FLD1-TAP-HIS5 ldb16::KANR*	This study
yPC4292	*MATa ura3Δ0 his3Δ1 leu2Δ0 met15Δ0 LDO16-GFP-KANR*	GFP collection
yPC4420	*MATa ura3Δ0 his3Δ1 leu2Δ0 met15Δ0 LDO16-3HA-HIS·fld1::NAT*	This study
yPC4622	*MATα ymr148w::HYGB ura3Δ0 his3Δ1 leu2Δ0 lys2Δ0 ERG6-mCherry-URA*	This study
yPC4649	*MATa ura3Δ0 his3Δ1 leu2Δ0 met15Δ0 tgl3::KANR*	KO collection
yPC4695	*MATa ura3Δ0 his3Δ1 leu2Δ0 met15Δ0 LDB16-TAP-HYGB fld1::NATR*	This study
yPC4847	*MATα ERG6-mCherry-URA NAT-CYC1-yeGFP-LDO45 lys2Δ0 ura3Δ0 his3Δ1 leu2Δ0*	This study
yPC4858	*MATa ura3Δ0 his3Δ1 leu2Δ0 met15Δ0 NAT-GAL1-LDO45*	This study
yPC4888	*MAT? ura3Δ0 his3Δ1 leu2Δ0 met15Δ0 NAT-GAL1-LDO45 ERG6-mCherry-URA*	This study
yPC4890	*MAT? ura3Δ0 his3Δ1 leu2Δ0 met15Δ0 NAT-GAL1-LDO45 fld1::HYGB*	This study
yPC4906	*MAT? ura3Δ0 his3Δ1 leu2Δ0 met15Δ0 NAT-GAL1- LDO45 ldb16::HYGB*	This study
yPC5042	*MATa ura3Δ0 his3Δ1 leu2Δ0 met15Δ0 PET10-mCherry-HIS*	This study
yPC5043	*MATa ura3Δ0 his3Δ1 leu2Δ0 met15Δ0 NAT-GAL1-LDO45 PET10-mCherry-HIS*	This study
yPC5045	*MATa ura3Δ0 his3Δ1 leu2Δ0 met15Δ0 TGL3-mCherry-HIS NAT-GAL1-LDO45*	This study
yPC5058	*MATa ura3Δ0 his3Δ1 leu2Δ0 met15Δ0 TGL3-mCherry-HIS*	This study
yPC5363	*MATa Ldb16-3xFLAG-HYGB leu2Δ0ura3Δ0his3Δ1met15Δ0*	This study
yPC7249	*MATa NAT-ADH1-DGA1-GFP-HIS2 leu2Δ0 met15Δ0 ura3Δ0 his3Δ1*	This study
yPC8693	*MATa ura3Δ0 his3Δ1 leu2Δ0 met15Δ0 PDR16-GFP-HIS3*	GFP collection
yPC9243	*MATa ura3Δ0 his3Δ1 leu2Δ0 met15Δ0 NAT-GAL1-LDO45 lro1::HIS*	This study
yPC9245	*MATa ura3Δ0 his3Δ1 leu2Δ0 met15Δ0 NAT-GAL1-LDO45 lro1::HIS dga1::KANR*	This study
yPC9247	*MATa ura3Δ0 his3Δ1 leu2Δ0 met15Δ0 NAT-GAL1-LDO45 dga1::KANR*	This study
yPC9530	*MATa NAT-GAL1-LDO45 KAN-ADH1-DGA1-GFP-HIS2 leu2Δ0 met15Δ0 ura3Δ0 his3Δ1*	This study
yPC9638	*MATa ura3Δ0 his3Δ1 leu2Δ0 met15Δ0 PDR16-GFP-HIS2 fld1::NAT*	This study
yPC9639	*MATa ura3Δ0 his3Δ1 leu2Δ0 met15Δ0 PDR16-GFP-HIS2 ymr147w-148w::KANR*	This study
yPC9658	*MATa ura3Δ0 his3Δ1 leu2Δ0 met15Δ0 LDO16-3HA-HIS3 ldb16::HYGB*	This study
yPC9695	*MATa ura3Δ0 his3Δ1 leu2Δ0 met15Δ0 NAT-GAL1-LDO45 PDR16-GFP-HIS*	This study
yPC9700	*MAT? ura3Δ0 his3Δ1 leu2Δ0 met15Δ0 PDR16-GFP-HIS ldb16::HYGB*	This study
yPC9702	*MAT? ura3Δ0 his3Δ1 leu2Δ0 met15Δ0 PDR16-GFP-HIS ldb16::HYGB ymr147w-148w::KANR*	This study
YPC9755	*MATa ura3Δ0 his3Δ1 leu2Δ0 met15Δ0 ymr147w::KANR PDR16-GFP-HIS*	This study
yPC9770	*MATa ura3Δ0 his3Δ1 leu2Δ0 met15Δ0 NAT-GAL1-LDO45 PDR16::KANR*	This study
YPC9782	*MAT? ura3Δ0 his3Δ1 leu2Δ0 met15Δ0 PDR16-GFP-HIS ymr147w-148w::KANR fld1::NAT*	This study
yPC9874	*MATa ura3Δ0 his3Δ1 leu2Δ0 met15Δ0 HIS-GAL-LDO16 pdr16::KANR*	This study
yPC9977	*MATa ura3Δ0 his3Δ1 leu2Δ0 met15Δ0 NAT-GPD-LDO45*	This study
yPC9978	*MATa ura3Δ0 his3Δ1 leu2Δ0 met15Δ0 NAT-GPD-LDO45 ldb16::HYGB*	This study
yPC9987	*MATa ura3Δ0 his3Δ1 leu2Δ0 met15Δ0 HIS-GAL1-LDO16 PDR16-GFP-KANR*	This study
yPC10248	*MATα ura3Δ0 his3Δ1 leu2Δ0 met15Δ0 dga1::NAT lro1::KANR*	This study
yPC10732	*MATa leu2Δ0 met15Δ0 ura3Δ0 his3Δ1 Vph1-GFP::HIS2*	This study
yPC10733	*MAT? leu2Δ0 met15Δ0 ura3Δ0 his3Δ1 Vph1-GFP::HIS2 ldb16::HYGB*	This study
yPC10734	*MAT? leu2Δ0 met15Δ0 ura3Δ0 his3Δ1 Vph1-GFP::HIS2 ymr147w-148w::KANR*	This study
yPC10735	*MAT? leu2Δ0 met15Δ0 ura3Δ0 his3Δ1 Vph1-GFP::HIS2 ymr147w::KANR*	This study
yPC10737	*MATa NAT-CYC1-yeGFP-LDO45 Sec63-Cherry-URA leu2Δ0 met15Δ0 ura3Δ0 his3Δ1*	This study
yPC10738	*MATa NAT-ADH1-DGA1-GFP-HIS2 KAN-GAL1-LDO16 leu2Δ0 met15Δ0 ura3Δ0 his3Δ1*	This study
yPC10739	*MATa Vph1-tdTomato-HIS leu2Δ0 met15Δ0 ura3Δ0 his3Δ1*	This study
yPC10740	*MAT? ymr147w::KANR Vph1-tdTomato-HIS leu2Δ0 his3Δ1 ura3Δ0*	This study
yPC10741	*MAT? ymr147w-ymr148w::KANR Vph1-tdTomato-HIS leu2Δ0 his3Δ1 ura3Δ0*	This study
yPC10742	*MATa ldb16::KANR Vph1-tdTomato-HIS leu2Δ0 his3Δ1 ura3Δ0*	This study
yPC10743	*MATa atg1::KANR Vph1-tdTomato-HIS leu2Δ0 his3Δ1 ura3Δ0*	This study

Table S2. **Primers used in this study**

Name	Sequence (5′–3′)	Purpose
Fld1F2	CATTTTTGTTAGAAAGGGTCAGGAAAAATCCAAGAAACATAGCGGTCGACGGATCCCCGGGTT	C-terminal tagging
Fld1R1	TAACAGCTAGGTTTTAAAATTATATAGCGAGAAGTACAATTTCGATGAATTCGAGCTCGTT	C-terminal tagging/deletion
Ldb16F2	CGGACATCGTTAATATAAAGATTTTACGAAGGAATTCTAGGGGTCGACGGATCCCCGGGTT	C-terminal tagging
Ldb16R1	TCTATCATTCACTTGTTAGTGCATGAGAAGAAGTAATTGCTCGATGAATTCGAGCTCGTT	C-terminal tagging/deletion
Ymr148wF1	GCCATTGGACTTGTTATTCCGTGTTCCCTACTTTTTTTGATACGGATCCCCGGGTTAATTAA	deletion
Ymr148wF2	GAGACTACTGCTAATAAAGCGGGTAATAAGTTCCAGCTCTCTGGTCGACGGATCCCCGGGTT	C-terminal tagging
Ymr148wR1	GACCTGCTAAACTTGCGAAAAATGTTTTTTTATTGCCGAGGTCGATGAATTCGAGCTCGTT	C-terminal tagging/deletion
Ymr147wF1	CGATTAAAATAAAAAGTGACATCTGAAAAACATCCAATACTCCGCGGATCCCCGGGTTAATTAA	deletion
Ymr147wR1	CTTTTAAATGCAGATCTGATTTTTTTTTCTATACTGTGCCTGTTCATCGATGAATTCGAGCTCGTT	deletion
Ymr148wGalpF4	CTTTGCCATTGGACTTGTTATTCCGTGTTCCCTACTTTTTTTGGAATTCGAGCTCGTTTAAAC	replace Ymr148wp by GAL1 promoter
Ymr148wGalpR2	GGTTAAGTAAACAAAAAAGAAAAATGTAGCGGTAGAAACCATTTTGAGATCCGGGTTTT	replace Ymr148wp by GAL1 promoter (no tag)
Ymr147wS1	GATTAAAATAAAAAGTGACATCTGAAAAACATCCAATACTCCGATGCGTACGCTGCAGGTCGAC	F primer for promoter replacement
YMR147wS4	ACCAAAAGGGACTTTTTCTTGTTATTCTTTCTACGATTCCTGGCTGCCATCGATGAATTCTCTGTCG	R primer for promoter replacement
Dga1 S1	CATATACATAAGGAAACGCAGAGGCATACAGTTTGAACAGTCACATAAATGCGTACGCTGCAGGTCGAC	F primer for promoter replacement
Dga1 S4	GCTTCCTTCTTCCTTCTTCCTTCTTCTTATATCATTGAATGTTCCTGACATCGATGAATTCTCTGTCG	R primer for promoter replacement
Erg6FCherry	CGCCGAAACCCCCTCCCAAACTTCCCAAGAAGCAACTCAAGACGGTGCTGGTTTAATTAAC	C-terminal tagging
Erg6RCherry	GTATATATCGTGCGCTTTATTTGAATCTTATTGATCTAGTGGAATTCGAGCTCGTTTAAAC	C-terminal tagging
Vph1FCherry	TGGAAGTCGCTGTTGCTAGTGCAAGCTCTTCCGCTTCAAGCGACGGTGCTGGTTTAATTAAC	C-terminal tagging
Vph1RCherry	GAAGTACTTAAATGTTTCGCTTTTTTTAAAAGTCCTCAAAATGAATTCGAGCTCGTTTAAAC	C-terminal tagging
Pet10FCherry	AGACCAAACCAATTCTAAGCCCGCGGCTGTGTCGACCAATGACGGTGCTGGTTTAATTAAC	C-terminal tagging
Pet10RCherry	GAAAAAAAATCAGGCGCACGATTAGCGCAAAACCAAATTAGAATTCGAGCTCGTTTAAAC	C-terminal tagging
Sec63FCherry	CGATACGGATACAGAAGCTGAAGATGATGAATCACCAGAAGACGGTGCTGGTTTAATTAAC	C-terminal tagging
Sec63RCherry	CGTCTAAGAGCTAAAATGAAAAACTATACTAATCACTTATATGAATTCGAGCTCGTTTAAAC	C-terminal tagging
Tgl3F Cherry	GAATTTAAATTAGACGACATAATAAGAGCAAGACGGAGTAGGGACGGTGCTGGTTTAATTAAC	C-terminal tagging
Tgl3R Cherry	CTATCAATAAAAAAAATAAGACAGAAAAAAGTGGAAACGATAGAATTCGAGCTCGTTTAAAC	C-terminal tagging
